# Enabling single qubit addressability in a molecular semiconductor comprising gold-supported organic radicals†
†Electronic supplementary information (ESI) available: Experimental procedures for synthesis, and physical and computational methods; electronic spectra; cyclic voltammetry; crystallographic figures; cw and pulsed EPR spectra and data analysis; orbital and spin density plots. CCDC 1857516 and 1857517. For ESI and crystallographic data in CIF or other electronic format see DOI: 10.1039/c8sc04500c


**DOI:** 10.1039/c8sc04500c

**Published:** 2018-11-22

**Authors:** Jake McGuire, Haralampos N. Miras, Emma Richards, Stephen Sproules

**Affiliations:** a WestCHEM School of Chemistry , University of Glasgow , Glasgow , G12 8QQ , UK . Email: stephen.sproules@glasgow.ac.uk; b School of Chemistry , Cardiff University , Main Building, Park Place , Cardiff , CF10 3AT , UK

## Abstract

A bis(dithiolene)gold complex is presented as a model for an organic molecular electron spin qubit attached to a metallic surface that acts as a conduit to electrically address the qubit. A two-membered electron transfer series is developed of the formula [Au^III^(adt)_2_]^1–/0^, where adt is a redox-active dithiolene ligand that is sequentially oxidized as the series is traversed while the central metal ion remains Au^III^ and steadfastly square planar. One-electron oxidation of diamagnetic [Au^III^(adt)_2_]^1–^ (**1**) produces an *S* = 1/2 charge-neutral complex, [Au^III^(adt_2_^3–^˙)] (**2**) which is spectroscopically and theoretically characterized with a near negligible Au contribution to the ground state. A phase memory time (*T*_M_) of 21 μs is recorded in 4 : 1 CS_2_/CCl_4_ at 10 K, which is the longest ever reported for a coordination complex possessing a third-row transition metal ion. With increasing temperature, *T*_M_ dramatically decreases becoming unmeasurable above 80 K as a consequence of the diminishing spin-lattice (*T*_1_) relaxation time fueled by spin–orbit coupling. These relaxation times are 1–2 orders of magnitude shorter for the solid dilution of **2** in isoelectronic [Ni(adt)_2_] because this material is a molecular semiconductor. Although the conducting properties of this material provide efficient pathways to dissipate the energy through the lattice, it can also be used to electrically address the paramagnetic dopant by tapping into the mild reduction potential to switch magnetism “on” and “off” in the gold complex without compromising the integrity of its structure. These results serve to highlight the need to consider all components of these spintronic assemblies.

## Abstract 2

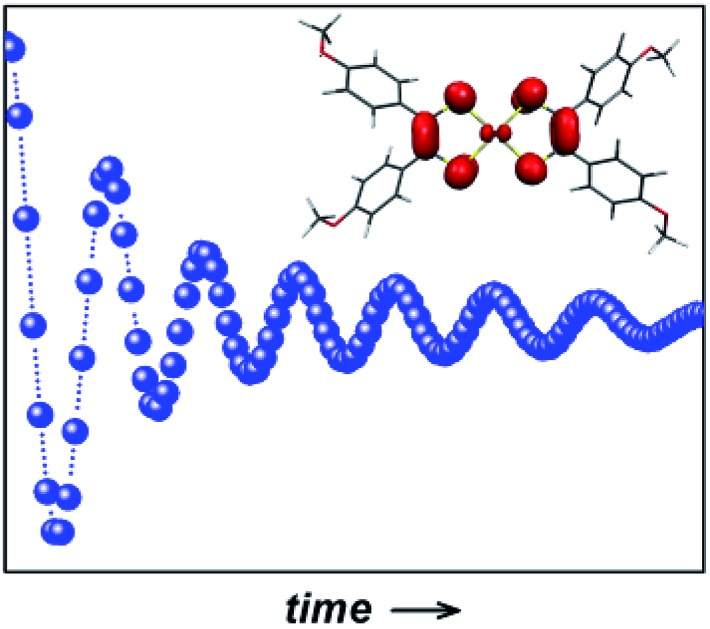
An organic radical attached to gold represents an electrically addressable prototype electron spin qubit with an impressively long coherence lifetime.

## Introduction

The emerging field of quantum information processing was borne out of the landmark paper by DiVincenzo which laid out the requirements for its elementary hardware – the qubit.^1^ These criteria have guided the development of many different qubit platforms such as photons,^2^ nuclear spins,^3^ and trapped ions.^4^ Electron spins are the most recent arrival, and have since garnered an enormous amount of attention packaged in solid-state materials such as semiconductors.^5^ These materials have the advantage of being scalable to give a very large number of qubits,^6^ and the electron spins they harbor are easily manipulated through magnetic or electric fields.^7^ These benefits come with the downside in that electron spins tend to have shorter coherence times, which is the lifetime of the superposition state often coined phase memory time, and DiVincenzo's third criterion. The best phase memory time would be provided by a completely isolated qubit, as it is the spins (electron and nuclear) in the surrounding environment that drive the loss of coherence. However this is impractical as without the ability to read and write information it can never perform any computation. The goal is to strike a balance between phase memory time while retaining the means to address the system in order to perform quantum logic.^8^ This is where chemistry makes its impact because molecules can be engineered that achieve the desired design requirements upon which the technology to run a quantum device can be trialled. For example, the intricacies of spin decoherence has greatly profited from examination of molecular species.^9^ In particular coordination complexes bearing a paramagnetic metal ion in an optimized ligand field have produced a bounty of detail about composition and structure, and in particular, the impact of nuclear spins on the spin dynamics, and therein the phase memory time.^10^–^16^ The result of these studies has driven phase memory times for coordination complexes to equal or even surpass the best among related matter spin qubits.^14^,^17^

Despite providing exceptionally long phase memory times, the vast majority of coordination complexes do not meet the design requirements for executing quantum gating that relies on addressing specific qubits or types of qubit to access superposition states needed to stage universal quantum logic. To tackle this goal, a new design is needed to produce molecules with multiple spin centers, and a mechanism in which they can be controlled. There have been a few molecular two-qubit systems that have been developed with this objective in mind,^18^ including prototypes that affect universal quantum logic.^19^ We proposed a new architecture of molecule-based spin qubits where the traditional role of the organic and inorganic components was inverted with spins residing on radical ligands and linked by diamagnetic metal ions. This was realized for a series of bis(dithiolene) complexes of group 10 metal ions of the formula [M(adt)_2_]^1–^ (M = Ni, Pd, Pt; adt^2–^ = bis(*p*-anisyl)-1,2-ethenedithiolate).^20^ The concept was expanded to a two-qubit species, [{Ni(adt)}_2_(μ-tpbz)]^2+^ (tbpz = 1,2,4,5-tetrakis(diphenylphosphino)benzene), where the terminal ligands are electrochemically oxidized to their dithiolene radical form. The phase memory time of 3.4 μs measured at 20 K is the longest reported for a metal-based molecular bipartite system. Moreover, the electrochemical trigger provides a means to selectively address the system as an applied potential can switch “on” and “off” the spin system.

Herein we present a study of the spin dynamics of square planar bis(dithiolene)gold complex, where the central Au(iii) d^8^ ion is diamagnetic and the unpaired electron is confined to the dithiolene ligands. This complex was selected for several reasons: (i) the charge-neutral state can facilitate for surface deposition by vacuum sublimation as a means to scale the system;^15^ (ii) the anisyl substituents on the dithiolene render the complex highly soluble in a range of esoteric solvents that have either no nuclear spins or nuclei with low magnetogyric ratios; (iii) the valence contribution to the electric field gradient (EFG) produces a colossal quadrupolar interaction that dwarfs the hyperfine interaction,^21^ permitting examination of the impact of quadrupolar coupling on spin dynamics; and (iv) there is near negligible metal contribution to the ground state,^22^ and thus considered an organic spin qubit. As such it is viewed as a model for an organic radical qubit bound to a gold surface,^23^ providing a unique opportunity to probe the effect of the materials that ultimately comprise quantum gates, where the gold represents the wiring that connects the spin qubit to the rest of the circuitry. In such a setup, the application of an applied potential would selectively address the redox state of the [Au(adt)_2_] molecules and electrically switch their spin “on” and “off”.^24^ The minuscule gold contribution to the ground state and the neutral charge delivers the longest phase memory time recorded for a third-row transition metal. However, the gold ion presents a heavy atom effect that prevents measurement above 80 K, and underscores the need to consider all components of the spintronic circuitry.

## Results and discussion

### Synthesis *via* characterization

Dialkyltin-protected dithiolenes have utility in transmetalation reactions that afford transition-metal dithiolene complexes and a cleaner synthesis compared to reactions employing the alkali-metal dithiolate salts.^25^ Dark green [PPh_4_][Au(adt)_2_] (**1**) is synthesized by the addition of two equivalents (adt)SnMe_2_ to potassium tetrachloroaurate in dichloromethane; the SnCl_2_Me_2_ by-product is conveniently washed away with MeOH. We find this synthetic approach consistently gives excellent yields (90%) and is decidedly preferable to the older P_4_S_10_/acyloin method devised by Schrauzer and Mayweg,^26^ at least with these more expensive noble metals.^21^,^27^

Complex **1** is diamagnetic as was judged from its ^1^H NMR spectrum, and its electronic spectrum displays two weak ligand field (LF) transitions in the visible region (Fig. S1†); no charge transfer (CT) bands are observed >800 nm in the near-infrared (NIR). Similar spectra have been reported for other diamagnetic, square planar Au^III^ complexes. Electrochemical measurements of **1** in CH_2_Cl_2_ solution containing 0.10 M [N(^*n*^Bu)_4_]PF_6_ revealed two reversible one-electron transfer waves at *E*_1/2_ = –0.143 V and *E*_1/2_ = +0.384 V, relative to the ferrocenium/ferrocene (Fc^+/0^) couple (Fig. S2†). The profile and reduction potentials are similar to related aryl-substituted dithiolenes of gold.^21^,^27^–^31^

The reaction of **1** with 0.5 equiv. of iodine in CH_2_Cl_2_ yielded dark brown crystals of [Au(adt)_2_] (**2**). This complex is paramagnetic as evinced by its room temperature magnetic moment of 1.72 *μ*_B_ (Evans method^32^) indicating an *S* = 1/2 ground state. The electronic spectrum displays a very intense absorption maximum in the NIR at 1556 nm (*ε* = 1.4 × 10^4^ M^–1^ cm^–1^) which has been previously assigned to an intervalence charge transfer (IVCT) transition of type [Au^III^(L)(L˙)] ↔ [Au^III^(L˙)(L)], which corresponds to a spin-allowed excitation from the highest doubly occupied molecular orbital (HOMO–1) to the singly occupied molecular orbital (SOMO) both of which are ligand-centered.^22^ This IVCT band is observed for all charge-neutral Au^III^ bis(dithiolene) complexes,^21^,^22^,^27^,^30^,^31^,^33^,^34^ and diagnostic of a coordinated π radical ligand. The longer wavelength for **2** reflects the ease with which adt is oxidized having softer, more polarizable sulfur atoms than its conjugated counterparts.^35^

### Crystallography

**Fig. 1:**
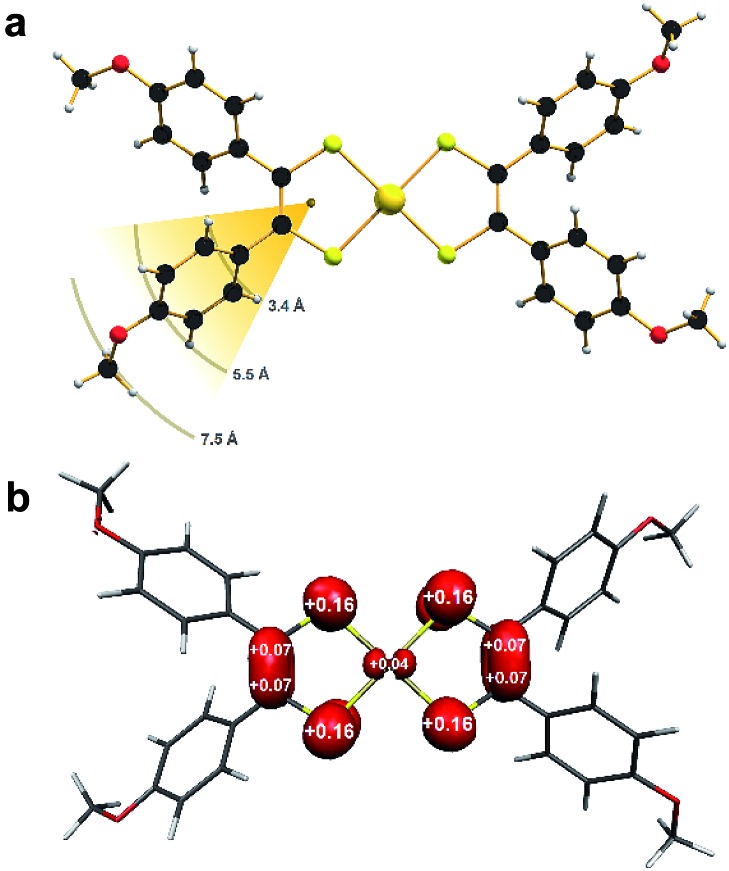
(a) The molecular structure of [Au(adt)_2_] in crystals of **2**, showing distance of ligand protons from the spin barycentre (color palette: Au, gold; S, canary; C, onyx; O, scarlet; H, alabaster). (b) Mulliken spin population analysis (red: α-spin).

Single-crystal X-ray diffraction revealed the anion in **1** to possess a near square planar {AuS_4_} core with a slight twist toward tetrahedral (*α* = 14.8°) ascribed to lattice packing (Fig. S3†). On the other hand, the coordination environment about the Au ion in **2** is perfectly planar (*α* = 0°; Fig. 1a).

The *p*-anisyl substituents are rotated relative to the {S_2_C_2_} plane at angles ranging 41–72° for both compounds. Therefore *via* induction, the anisyl group is electron donating reflecting the softer, more polarizable sulfur donor atoms in this ligand. An important consideration relating to the spin dynamics of this molecular spin qubit are the protons on the *p*-anisyl substituents of the dithiolene. Despite the absence of conjugation which ensures the spin density is confined to the {S_2_C_2_} core (Fig. 1b), these protons nevertheless present an efficient decoherence pathway through dipolar coupling.^11^ The three types of proton in the ligand – two aromatic and one methyl – are on average 3.4 Å, 5.5 Å and 7.5 Å, respectively, away from the spin locus (Fig. 1a). The ligand oxidation level is revealed in the intraligand bond distances for **2** compared with those in **1**. The average S–C bond distance of 1.739 ± 0.002 Å and average C–C distance of 1.375 ± 0.003 Å are shorter and longer, respectively, than the corresponding bond lengths in the dianionic dithiolate form of the ligand in **1** at 1.767 ± 0.002 Å and 1.351 ± 0.003 Å, respectively (Table 1). This is characteristic of an open-shell dithiolene radical, which due to inversion symmetry, is distributed over both ligands with an electronic structure defined as [Au^III^(adt_2_^3–^˙)]^0^.^36^ Therefore, the metal ion is +III in both as evinced by the similarity of the average Au–S bond lengths of 2.3165 ± 0.0009 Å in **1** and 2.3006 ± 0.0009 Å in **2**. The observed intraligand metrics are in excellent agreement with a number of monoanionic and neutral aryl-substituted bis(dithiolene)gold complexes.^21^,^27^,^28^,^31^

**Table 1:** Comparison of experimental and calculated metrics^*a*^

	**1**		**2**	
	Exptl	Calcd	Exptl	Calcd
Avg. Au–S	2.3165(9)	2.350	2.3006(9)	2.335
Avg. S–C	1.767(2)	1.778	1.739(2)	1.747
Avg. C–C	1.351(3)	1.363	1.375(3)	1.384
Avg. S–Au–S	89.18(3)	87.8	88.81(2)	87.7
*α* ^*b*^	14.8	2.7	0.0	0.4

^*a*^Distances in angstrom; angles in degrees.

^*b*^Dihedral angle between mean AuS_2_ planes.

### Continuous-wave EPR spectroscopy

**Fig. 2:**
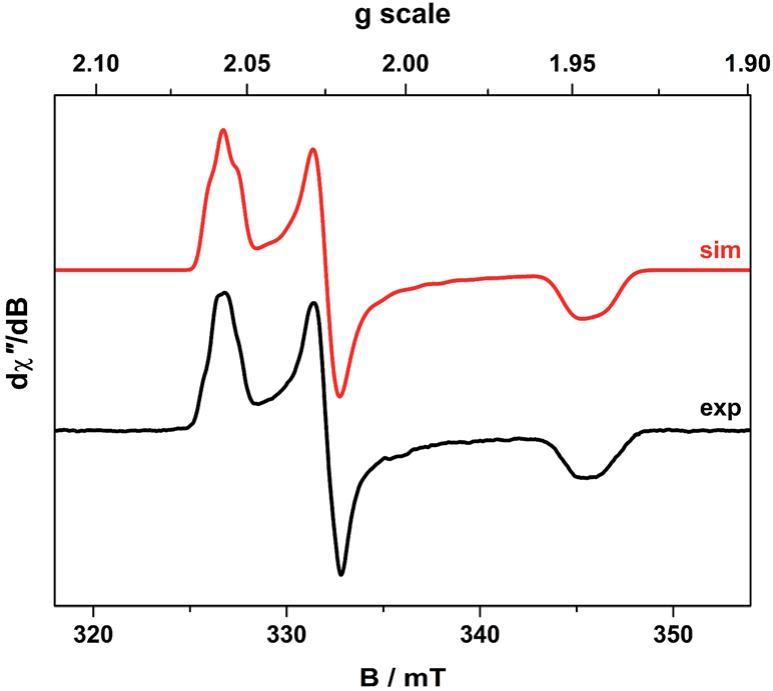
X-band EPR spectrum of **2** recorded in THF at 130 K (experimental conditions: frequency, 9.4098 GHz; power, 0.63 mW; modulation, 0.3 mT). Experimental data are represented by the black line; simulation is depicted by the red trace.

The cw X-band EPR spectrum of **2** recorded in THF at 130 K display a signal typical of an *S* = 1/2 system with rhombic *g*-values similar to related compounds (Fig. 2).^21^,^27^,^33^,^34^,^37^ The splitting pattern (*g*_*y*_ > *g*_*x*_ > *g*_e_ > *g*_*z*_) is the same as observed for isoelectronic bis(dithiolene) monoanions of group 10 metals given an identical ^2^B_2g_ ground state (*vide infra*).^20^,^22^ The spectrum exhibits a remarkable hyperfine splitting from the ^197^Au nucleus (*I* = 3/2, 100% abundant), where the quartet splitting of each principal *g*-value manifests with an unusual spacing and intensity distribution of the hyperfine lines. This outcome is caused by a sizeable EFG at the ^197^Au nucleus that produces the strong quadrupole interaction whose principal axes are orientated in a different direction from those of the *g* and *A* matrices. The misalignment of the principal quantization axes leads to mixing of hyperfine levels and emergence of forbidden (Δ*m_I_* ≠ 0) transitions in the EPR spectrum. This unique situation, where the quadrupole interaction is larger than the magnetic hyperfine interaction, can only arise if the spin is located on the ligand coordinated to a Au^III^ ion with a (d_*xz*_,_*yz*_)^4^(d_*z*^2^_)^2^(d_*xy*_)^2^(d_*x*^2^–*y*^2^_)^0^ electronic configuration.

This generates the large valence contribution to the EFG producing the dominant quadrupole interaction seen in the spectrum. A similar scenario was revealed for neutral tris(dithiolene)rhenium species whose electronic structure was unambiguously defined from its truly exceptional EPR spectrum.^38^ An excellent fit was obtained with the *g*-, *A*-, and *P*-values listed in Table 2. Minor details such as the relative rotations of the different principal axes could not be resolved. Each *A*-value must have the same sign as inferred from the close match of the isotropic value to the average from the anisotropic values. The assignment as negative was derived from the ^197^Au nuclear *g*-value assuming a dominant Fermi contact contribution, which is nicely corroborated by DFT calculations (*vide infra*).

**Table 2:** Summary of experimental and calculated spin-hamiltonian parameters for **2**

Parameter	Experimental	Calculated^*a*^
*g*_iso_	2.0094	
*g*_*x*_	2.0245	2.0294
*g*_*y*_	2.0575	2.0616
*g*_*z*_	1.9450	1.9493
〈*g*〉^*b*^	2.0090	2.0134
*R_g_* ^*c*^	0.29	0.29
Δ*g*^*d*^	0.1125	0.1123
*A*_iso_ ^*e*^	–5.0	
*A*_*x*_ ^*e*^	–3.5	–4.5
*A*_*y*_ ^*e*^	–7.0	–4.5
*A*_*z*_ ^*e*^	–6.5	–4.7
〈*A*〉^*e*^ ^,^^*f*^	–5.7	–4.6
*P* ^*e*^ ^,^ ^*g*^	–150	
*η* ^*e*^ ^,^ ^*h*^	–50	

^*a*^From ZORA-PBE0 DFT calculations.

^*b*^〈*g*〉 = ( = (*g*_*x*_ + *g*_*y*_ + *g*_*z*_)/3 ≈ *g*_iso_.

^*c*^Rhombicity, *R*_g_ = (*g*_*y*_ – *g*_*x*_)/(*g*_*y*_ – *g*_*z*_).

^*d*^ *g*-anisotropy, Δ*g* = *g*_*y*_ – *g*_*z*_.

^*e*^In units 10^–4^ cm^–1^.

^*f*^〈*A*〉 = ( = (*A*_*x*_ + *A*_*y*_ + *A*_*z*_)/3 ≈ *A*_iso_.

^*g*^ *P* = [*P*_*z*_ – (*P*_*x*_ + *P*_*y*_)/2]/3.

^*h*^ *η* = (*P*_*x*_ – *P*_*y*_)/2.

### Theoretical calculations

The geometry-optimized structures for the complex anion in **1** and neutral **2** are in excellent agreement with the experimental data with both the Au–S and intraligand bond distances and angles accurately reproduced (Table 1). Moreover the optimized structures are strictly planar demonstrating the slight tetrahedralization about the Au ion in **1** is a consequence of crystal packing. Inspection of the frontier MOs reveals four metal d orbitals at deeper binding energies than the ligand-based b_3g_ and b_2g_ (*D*_2*h*_ point group) which undergo symmetry-allowed π interactions with metal d orbitals.^36^ In both, the HOMO is the b_2g_ symmetric ligand-centered orbital, which is doubly occupied in **1** leading to its assignment as [Au^III^(adt)_2_]^1–^. As the redox-active orbital, oxidation of **1** gives the b_2g_ SOMO in **2**, and an electronic structure defined as [Au^III^(adt_2_^3–^˙)]^0^ (Fig. S6†).^22^ This is consistent with the spin population distribution where the unpaired spin is delocalized over both ligands with miniscule spin residing at the Au^III^ center (Fig. 1b). The electronic structure of **2** has been verified by very accurate calculation of the *g*- and *A*-values (Table 1). This level of precision allows for meaningful insight that correlates composition and electronic structure factors on the spin dynamics of molecular qubits based on coordination complexes.

### Pulsed EPR spectroscopy

The spin relaxation properties as parameterized by spin-lattice (*T*_1_) and phase memory (*T*_M_) lifetimes were investigated for **2** at field positions corresponding to the most intense resonance lines. The advantage of a charge-neutral molecular qubit with bulky *p*-anisyl substituents allowed us to explore a range of esoteric solvent mixtures. The selection included chloroform-*d* (CDCl_3_), carbon tetrachloride (CCl_4_), carbon disulfide (CS_2_) and trichloroacetonitrile (Cl_3_CCN), whose constituent atoms have weak magnetogyric ratios (^2^H = 4.11 × 10^–7^; ^14^N = 1.93 × 10^–7^; ^35^Cl = 2.62 × 10^–7^; ^37^Cl = 2.17 × 10^–7^ T^–1^ s^–1^) which are an order of magnitude smaller than for ^1^H. In addition, the solvents contain no methyl functionality – CH_3_ or CD_3_ – whose rotation provide an especially efficient decoherence pathway even at the lowest temperatures.^39^ In order to achieve a good frozen glass, chlorinated solvents were combined with Cl_3_CCN as a 4 : 1 mixture. This ratio was inverted for the Cl_3_CCN and CS_2_ glassing mixtures in combination with CCl_4_. In contrast to the recent study of isoelectronic complexes, [PPh_4_][M(adt)_2_] (M = Ni, Pd, Pt) in CD_2_Cl_2_/DMF-*d*_7_,^20^ here the only protons and methyl groups are those on the periphery of the dithiolene ligand (Fig. 1a).

**Fig. 3:**
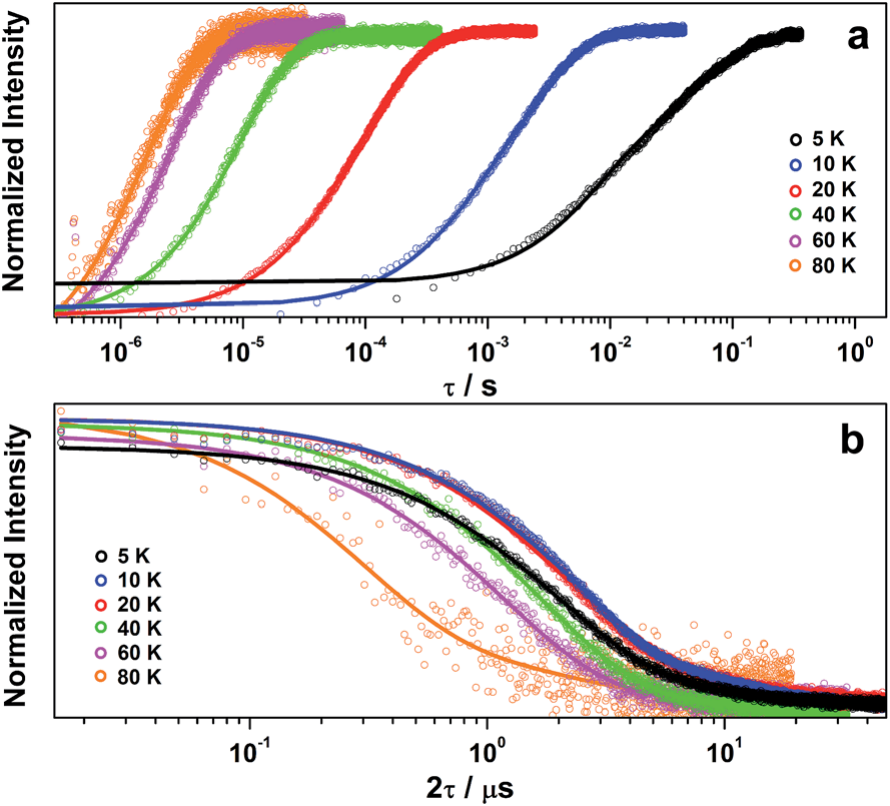
Temperature dependence of (a) inversion recovery and (b) Hahn echo decay for **2** in 4 : 1 CCl_4_/Cl_3_CCN. Experimental data are represented by open circles and corresponding exponential fit depicted by the line.

**Fig. 4:**
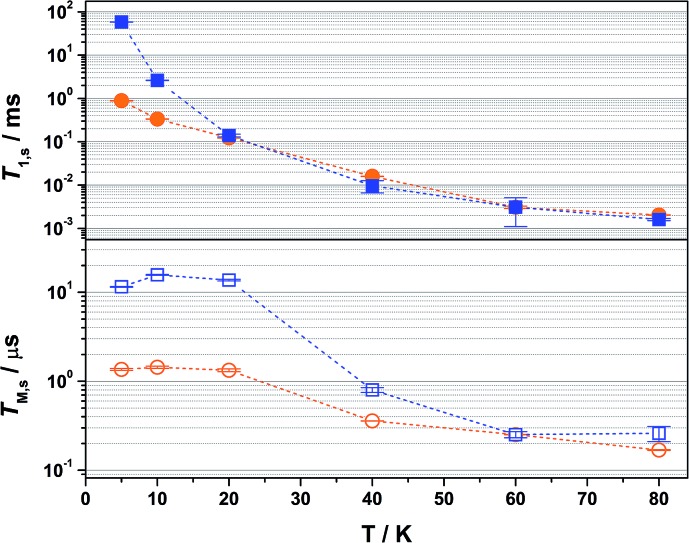
Comparison of the temperature dependence of *T*_1,s_ (top) and *T*_M,s_ (bottom) relaxation times for **2** diluted in 4 : 1 CCl_4_/Cl_3_CCN (blue filled and open squares) and 2% in [Ni(adt)_2_] (orange filled and open circles) over the range 5–80 K. Error bars are based on the standard deviation of the fit.

Inversion recovery data were collected on a solution of **2** in 4 : 1 CCl_4_/Cl_3_CCN to assess the temperature dependence of the spin-lattice relaxation between 5 and 80 K (Fig. 3a). The curves are modelled with a biexponential function that yielded values for the fast (*T*_1,f_) and slow (*T*_1,s_) relaxation processes, where the former is attributed to spectral diffusion while the latter is assigned the signature spin-lattice relaxation time (Fig. 4). The biexponential fit was only applied up to 20 K; beyond this temperature the fast process merged with the spectral noise, and a monoexponential decay curve is sufficient to estimate the slow process until it becomes irretrievable above 80 K.

Overall the *T*_1,s_ decreases exponentially from 58.3 ms at 5 K to 1.6 ms at 80 K. There is a slight orientation dependence with the longest time recorded for *B*_0_ = 375.7 mT (*g*_*z*_) approximately 12% greater than at the other principal *g*-values (Table S6†). It should be noted that the quadrupole coupling is weakest around *g*_*z*_,^21^ which indicates quadrupolar coupling does attenuate spin relaxations times. The steep decline in the spin-lattice relaxation time is a consequence of the large spin–orbit coupling (SOC) constant of Au at ∼4500 cm^–1^. Below 10 K, a direct spin relaxation process is dominant,^40^ but as the temperature increases the Raman mechanism takes precedence,^41^ and becomes more efficient with increasing SOC.^12^ The significance of SOC has been previously shown to impact on spin-lattice times when comparing first- and second-row metals in systems where the metal is the spin host.^42^ We recently revealed for this system where the ligand is the spin host anchored by the metal ion, that the latter represents a heavy-atom effect.^20^ The phenomenon is particularly pervasive in this qubit design, and we have begun to explore alternative uses for these molecules to replace dichalcogenides in graphene-based heterostructures.^43^

**Fig. 5:**
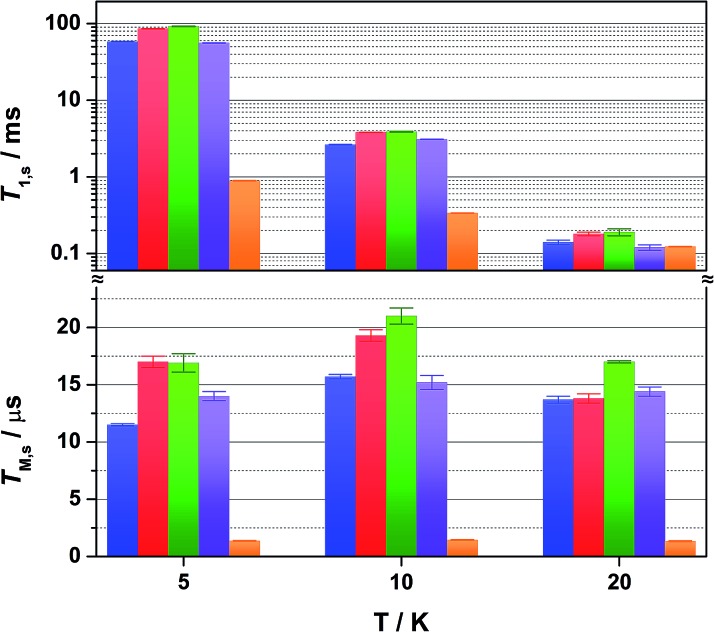
Comparison of the temperature dependence of *T*_1,s_ (top) and *T*_M,s_ (bottom) for 1 mM solutions of **2** in 4 : 1 CCl_4_/Cl_3_CCN (blue), 4 : 1 CDCl_3_/Cl_3_CCN (red), 4 : 1 CS_2_/CCl_4_ (green), 4 : 1 Cl_3_CCN/CCl_4_ (violet), and the polycrystalline material (2% in [Ni(adt)_2_], orange) at 5, 10 and 20 K. Error bars are based on the standard deviation of the fit.

Altering the solvent medium had a noticeable impact on *T*_1,s_, as gauged from measurements at 5, 10 and 20 K (Fig. 5). Of the four solvent mixtures tested, the longest time was recorded at 5 K for 4 : 1 CS_2_/CCl_4_ of 92 ms – the combination with the least spin-active nuclei. Slightly shorter times were provided for 4 : 1 CDCl_3_/Cl_3_CCN at 86 ms, ahead of 4 : 1 CCl_4_/Cl_3_CCN at 58 ms and 4 : 1 Cl_3_CCN/CCl_4_ at 56 ms. These lifetimes are among some of the longest recorded for molecular electron spin qubits.^9^ Moreover, they an order of magnitude longer than their isoelectronic group 10 counterparts, [M(adt)_2_]^1–^ (M = Ni, Pd, Pt),^20^ and related Ni bis(dithiolenes) reported by Bader *et al.*^16^ This highlights the importance of complex charge – **2** being neutral – and the significantly smaller contribution from Au to the magnetic orbital compared with the group 10 analogues.

The most interesting result is supplied by the solid dilution of **2** in the diamagnetic, charge-neutral [Ni(adt)_2_]. As Atzori *et al.* have shown that 10% dilution of the paramagnetic species in the diamagnetic analogue gave essentially the same relaxation times as the 0.1% dilution,^44^ we have used 2% dilution in order to give a sufficiently intense Hahn-echo so that the number of averages (scans) was equivalent to the frozen solution samples. The spin-lattice relaxation time measured at 5 K is 0.89 ms, two orders of magnitude smaller than that recorded in frozen solution at an equivalent temperature (Fig. 4). At 20 K and above, the *T*_1,s_ lifetimes for [Au_0.02_Ni_0.98_(adt)_2_] match the frozen solution data, and run parallel up to the highest measurement temperature of 80 K. This profile is borne out of the planar structure of **2** and its diamagnetic host. Neutral gold bis(dithiolenes) are single component semiconductors,^27^,^33^,^45^,^46^ and the conductivity is tuned when doped into the corresponding neutral Ni complex.^29^ The planar molecules stack into dimerized columns with intermolecular distances as short as 3.6 Å, which is the source of the observed singlet-triplet magnetic behaviour in neutral gold bis(dithiolenes).^29^,^46^

As detailed by Fourmigué and co-workers, dilution of **2** in [Ni(adt)_2_] will give rise to dimers of **2** embedded uniformly in the diamagnetic matrix, where the conductivity derives from tunnelling between gold dimer fragments either along the chain or perpendicular to it. Interestingly, the EPR spectrum of polycrystalline **2** is identical to the frozen solution spectrum rather than a spin-triplet signal from a dimer moiety (Fig. S24†), and may suggest that at the low concentration used here (2%), the Ni analogue may disrupt the dimerization. The short intermolecular distances within and between chains that give rise to the semiconducting properties also serve as an efficient pathway for spin-lattice relaxation. This is only noticeable <20 K when compared to frozen solution data (Fig. 4), as above this temperature the SOC-driven Raman mechanism is dominant and is less dependent on intermolecular interactions.

The decay of the Hahn echo measured at the magnetic field corresponding to the absorption maxima (*g*_*x*_) in the EPR spectrum follows a biexponential profile; the temperature dependence for **2** in 4 : 1 CCl_4_/Cl_3_CCN is shown in Fig. 3b. The fit gives an estimate for the fast (*T*_M,f_) and slow (*T*_M,s_) relaxation processes, with the latter defined as the phase memory time when measuring qubit performance. An exceedingly long phase memory time of 15.6 μs is recorded for **2** at 10 K, and this increases to 17.6 μs when the field position is shift to 375.7 mT corresponding to *g*_*z*_, an increase of 12% which aligns with the orientation dependence observed for *T*_1,s_ (*vide supra*). This time betters many recently reported *S* = 1/2 coordination complexes.^9^ The few that surpass this time have had their composition and environment rigorously engineered to be devoid of nuclear spins.^13^,^14^,^16^ Moreover, this is 4–5 times longer than phase memory times reported for any other second- or third-row transition metal.^12^,^47^ The phase memory time is improved by altering the solvent mixture, reaching a maximum of 21 μs in 4 : 1 CS_2_/CCl_4_ – the medium with the fewest nuclei spins (Fig. 5). The major contributor to spin decoherence are electron–nuclear spin interactions which is the dominant factor at very low temperatures (<30 K). The nuclear spin bath is limited to the protons on the anisyl substituents of the dithiolene ligand; low magnetogyric ratios for ^35,37^Cl nuclei in the solvent and ^195^Au ensures their contribution is negligible. The pitch of the anisyl substituents to a non-conjugated orientation relative to the dithiolene core ensures ^1^H interaction is dipolar and governed by the interspin distance. Here with the locus of the spin on the ligand, only the methoxy groups lie beyond the spin-diffusion barrier (Fig. 1a).^11^ The distribution of spin density away from the metal ion and the disposition of the SOMO orthogonal to the plane of the complex facilitates greater interaction with the solvent medium. There is a slightly stronger interaction between the more polar Cl_3_CCN and the electronegative {S_2_C_2_} core of the dithiolene ligand as evinced by the relaxation times (Fig. 5).

There is an overall increase in the relaxation rate with increasing temperature, though the shorter *T*_M,s_ at 5 K than observed at 10 K is due to a loss of solubility leading to inhomogeneity in the glass. The swift decline above 20 K is driven by a comparable reduction in the spin-lattice relaxation time which is the ultimate limit for *T*_M,s_,^48^ where spin-lattice and spin–spin relaxation approach parity, preventing measurement of the Hahn echo decay above 80 K (Fig. 4). The solid dilution of **2** in [Ni(adt)_2_] afforded the shortest *T*_M,s_ of 1.44 μs at 10 K, an order of magnitude smaller than for the frozen solution samples (Fig. 4). As *T*_1,s_ is sufficiently long at 10 K, the short phase memory time is a consequence of the greater population of protons in the spin bath as the dithiolene ligand in the diamagnetic host is fully protiated and the efficient stacking in the solid state brings these decohering spins much closer to the electron spin on the gold complex.

**Fig. 6:**
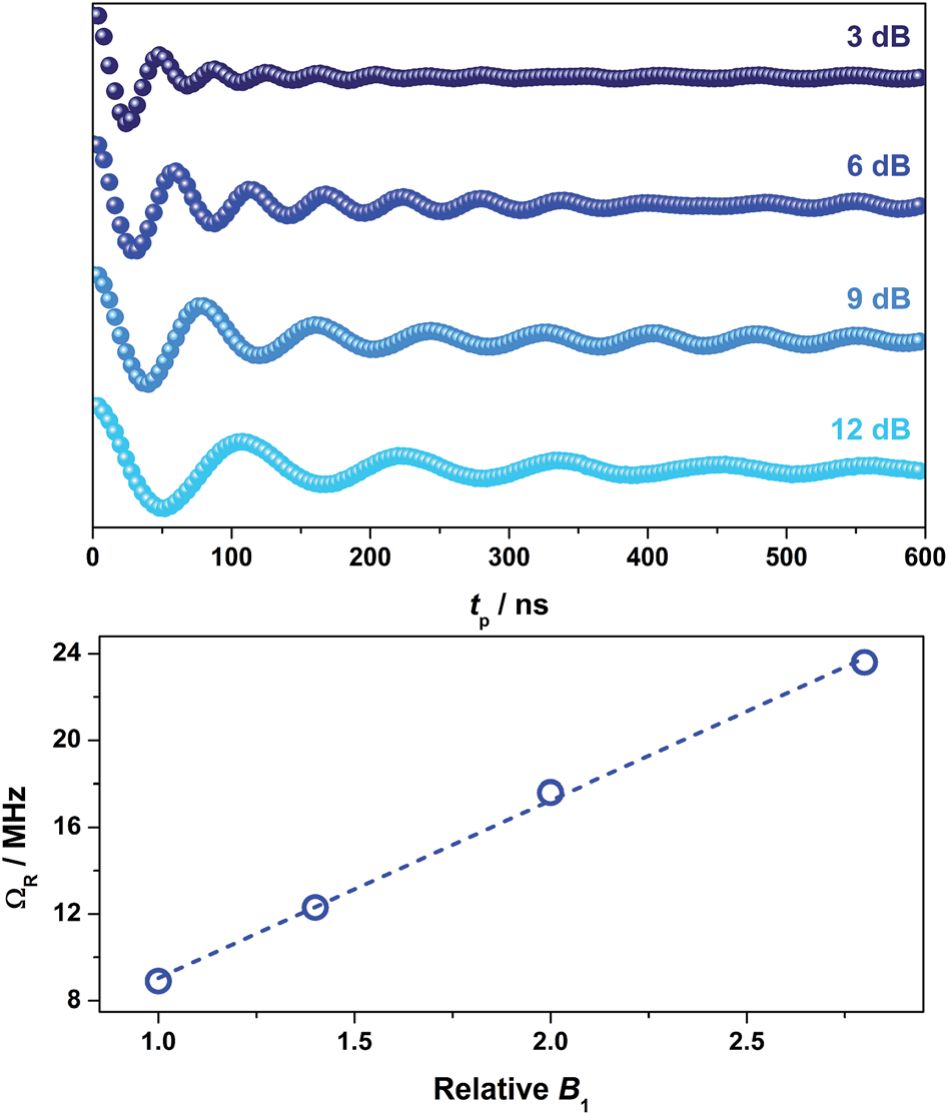
Variable power nutation measurements for **2** in 4 : 1 CCl_4_/Cl_3_CCN at 10 K and 343.5 mT (top), and linear dependence of Ω_R_ with respect to the *B*_1_ field (bottom). Dashed trace represents line of best fit.

To demonstrate coherent spin control, echo-detected nutation experiments were performed by applying a microwave pulse of duration *t*_p_ to produce Rabi-like oscillations between two states that correspond to arbitrary superpositions of the electron spin (Fig. 6). The physical origin is confirmed by the linear dependence of the oscillation frequency (Ω_R_) with the applied microwave amplitude (*B*_1_), which was varied by selecting microwave attenuations of 3, 6, 9 and 12 dB (Fig. 6). Changes in the oscillations were observed at *t*_p_ > 400 ns that derive from interaction with ligand protons and are independent of the microwave attenuation.^49^ The glassing medium had no bearing on the Rabi frequency with all values within experimental error (Fig. S28†). The short phase memory time recorded on **2** diluted 2% in [Ni(adt)_2_] similarly leads to short Rabi frequencies (Fig. S29†). The nutation data measured at 6, 9 and 12 dB gave values with the expected linear dependence on the microwave amplitude. However, at 3 dB, the peak is masked in the Fourier transform by features deriving from ^1^H hyperfine coupling (Fig. S29†). Nevertheless, the Rabi frequency at 3 dB microwave attenuation is estimated at 7.1 MHz by extrapolation of the linear fit from the plot of Ω_R_*versus B*_1_ (Fig. S29†).

## Conclusion and outlook

The use of a ligand radical spin host in charge-neutral [Au(adt)_2_] gave an impressive phase memory time of up to 21 μs, which is significantly longer than other qubits based on second- and third-row metals. This lifetime is comparable to the current state of the art in molecule-based systems. The performance of this Au complex when compared to isoelectronic group 10 species,^20^ derives from the combination of the complex charge and the miniscule contribution to the ground state from the Au 5d orbitals. The neutral charge allowed for testing of a range of unusual solvents comprised of nuclei with low magnetogyric ratios which had not been used in the study of spin dynamics previously. The near negligible Au contribution to the ground state materializes in the small hyperfine coupling that is dwarfed by the nuclear quadrupolar coupling, and there is an indication from the orientation dependent measurements that quadrupolar interactions also serve to diminish the lifetime of the cohered state. In conjunction with these nuclear characteristics, the colossal SOC supplied by the Au ion precluded measurement above 80 K, following the trend established for [M(adt)_2_]^1–^ (M = Ni, Pd, Pt), as SOC amplifies the Raman process that accelerates spin-lattice relaxation above 20 K.

Relaxation times are markedly shorter for the solid dilution of **2** in the isoelectronic nickel complex on account of the semiconducting properties of the doped mixture. This greatly impacts spin-lattice relaxation, which in turn shortens the phase memory time though this is still 1.44 μs at 10 K. The conductivity could provide a unique handle in tackling the next stage in the DiVincenzo criteria,^1^ namely single qubit addressability. Given the persistent square planar geometry adopted by each member of this electron transfer series (monocationic and monoanionic species, *S* = 0; neutral complex, *S* = 1/2) there is no disruption to the stacked structure of the doped material when a potential is applied that can switch the magnetism “on” and “off”, and therein the ability to switch between various spin states and entanglement scenarios. We will continue to develop this ligand radical platform with the aim of executing electrically operated multi-qubit quantum gates using molecular semiconducting assemblies.

## Conflicts of interest

There are no conflicts to declare.

## Supplementary Material

Supplementary information

Crystal structure data
