# Supplementary material for: Enabling single qubit addressability in a molecular semiconductor comprising gold-supported organic radicals
Source: Chem Sci. 2018 Nov 22;10(5):1483–91. doi: 10.1039/c8sc04500c (PMC6354843; doi:10.1039/c8sc04500c)
Supplement: Supplementary file 1 [file SC-010-C8SC04500C-s001.pdf]

Electronic Supplementary Material (ESI) for Chemical Science.
This journal is © The Royal Society of Chemistry 2018

Supporting Information

Enabling Single Qubit Addressability in a Molecular Semiconductor Comprising Gold-Supported Organic Radicals

Jake McGuire,[a] *Haralampos N. Miras,*[a] *Emma Richards*[b] *and Stephen Sproules**[a]

[a] WestCHEM School of Chemistry, University of Glasgow, Glasgow G12 8QQ (UK)

[b] School of Chemistry, Cardiff University, Main Building, Park Place, Cardiff CF10 3AT (UK)

Email: stephen.sproules@glasgow.ac.uk

Experimental Section

Synthesis. The compound (adt)$SnMe_2$ was prepared following the published method.[1] Solvents were either dried with a system of drying columns (CH_2Cl_2, MeCN) or degassed by five successive freeze-pump-thaw cycles and dried over 3 Å molecular sieves ($CDCl_3$, CCl_4, CS_2, Cl_3CCN). All other reagents were used as received.

Synthesis of [PPh_4][Au(adt)$_2$] (1). A 50 mL Schlenk flask charged with K[$AuCl_4$] (50 mg; 0.132 mmol), (adt)$SnMe_2$ (120 mg; 0.267 mmol) and MeCN (20 mL), and stirred at ambient temperature for 1 h. To the dark green reaction mixture was added PPh_4Br (56 mg; 0.132 mmol) in a single portion under a flow of nitrogen, and mixture further stirred for 30 min. The solvent was removed under reduced pressure and the resultant solid was washed with H_2O (3 × 10 mL), MeOH (3 × 10 mL), and Et_2O (3 × 10 mL), and dried under vacuum. Yield: 135 mg (90%). Anal. Calcd for $C_{56}H_{48}O_4PS_4Au·0.5CH_2Cl_2$: C, 57.33; H, 4.17. Found: C, 57.08; H, 3.95. ^{1}H NMR (MeCN-d_3; δ / ppm): 7.94 (t, 4H, Ph), 7.73 (m, 20H, PPh_4^+), 7.01 (d, 8H, J_{HH} = 8.5 Hz, Ph), 6.55 (d, 8H, J_{HH} = 8.5 Hz, Ph), 3.76 (s, 12 H, -OCH_3). ^{13}C NMR (DMSO-d_6; δ / ppm): 177.40 (s), 157.99 (s), 135.84 (s), 135.10 (s), 134.99 (s), 131.00 (s), 130.87 (s), 113.49 (s), 55.36 (s). Absorption spectrum (CH_2Cl_2; λ_{max} / nm (ε / 10^{-1} M^{-1} cm^{-1})): 472 (0.32), 696 (0.16). ESI mass spectrum (neg. ion): *m/z* 801.04 $[M]^-$.

Synthesis of [Au(adt)$_2$] (2). To a 50 mL Schlenk flask containing **1** (130 mg; 0.114 mmol) in CH_2Cl_2 (20 mL) was added I_2 (29 mg; 0.114 mmol) left to stir at ambient temperature for 1 h. The initial dark green reaction mixture rapidly transitioned to dark brown solution. The solvent was stripped under reduced pressure and the residue washed with H_2O (3 × 10 mL), MeOH (3 × 10 mL), and Et_2O (3 × 10 mL), and dried under vacuum. Yield: 76 mg (83%). Anal. Calcd for $C_{32}H_{28}O_4S_4Au$: C, 47.90; H, 3.50. Found: C, 47.50; H, 3.45. Absorption spectrum (CH_2Cl_2; λ_{max} / nm (ε / 10^{-4} M^{-1} cm^{-1})): 455 (0.38), 557 (sh, 0.18), 633 (sh, 0.13), 1556 (1.38). ESI mass spectrum (neg. ion): *m/z* 801.04 $[M]^-$.

X-ray Crystallographic Data Collection and Structure Refinement. Diffraction quality crystals of **1** and **2** were obtained by slow diffusion of hexanes into a concentrated dichloromethane solution of the complex. The crystals were coated with paratone oil and mounted on the end of a nylon loop attached to the end of the goniometer. Data were collected with a Bruker SMART APEX CCD diffractometer equipped with a Kryoflex attachment supplying a nitrogen stream at 150 K. Structure solution and refinement were carried out with SHELXS-97[2] and SHELXL-97[3] using the WinGX[4] software package. Corrections for incident and diffracted beam absorption effects were applied using empirical absorption corrections.[5] For **1**, all non–hydrogen atoms were refined with anisotropic thermal parameters and the positions of hydrogen atoms of the PPh_4^+ counterion were calculated based on stereochemical considerations and refined isotropically. For **2**, all non-hydrogen atoms were refined using anisotropic thermal parameters. Additionally, a severely disordered CH_2Cl_2 solvent molecule was refined isotropically, and split over two positions based on the electron density identified by the DF map. Final unit cell data and refinement statistics are collected in Table S1. The crystallographic data for **1** and **2** (CCDC 1857516 and 1857517) can be obtained free of charge from the Cambridge Crystallographic Data Centre, 12, Union Road, Cambridge CB2 1EZ; fax:(+44) 1223- 336-033, deposit@ccdc.cam.ac.uk.

Table S1 Crystallographic Data

compound	$PPh_4[Au(adt)_2]$	$[Au(adt)_2]\cdot CH_2Cl_2$
formula	$C_{56}H_{48}O_4PS_4Au$	$C_{33}H_{30}O_4S_4Cl_2Au$
fw	1141.12	884.66
T, K	150(2)	150(2)
λ, Å	0.71073	0.71073
2θ range, deg	4.35 – 52.28	4.46 – 53.16
crystal system	triclinic	monoclinic
space group	$P\bar{1}$	$P2_1/c$
a, Å	11.913(5)	11.584(4)
b, Å	13.949(5)	15.448(5)
c, Å	15.859(6)	9.441(3)
α, deg	81.424(4)	90
β, deg	72.882(4)	102.511(2)
γ, deg	79.945(4)	90
V, Å^3	2467(2)	1649.5(9)
Z	2	2
ρ, g cm^{-3}	1.536	1.781
μ, mm^{-1}	3.231	4.913
crystal size	0.08 × 0.12 × 0.13	0.08 × 0.10 × 0.20
color, habit	green block	brown block
reflections collected	27552	21212
independent data	9442	3449
restraints	0	0
parameters refined	595	216
GoF^{a}	1.169	1.062
R1,^{b,c} wR2^{d,c}	0.0151, 0.0427	0.0187, 0.0463
R1,^{b,e} wR2^{d,e}	0.0163, 0.0495	0.0202, 0.0536
largest diff. peak, e Å^{-3}	0.375	1.610
largest diff. hole, e Å^{-3}	-1.020	-0.990

^{a} GoF = $\{\Sigma[w(F_o^2 - F_c^2)^2]/(n - p)\}^{1/2}$, where n = number of reflections and p is the total number of parameters refined. ^{b} R1 = $\Sigma||F_o| - |F_c||/\Sigma|F_o|$. ^{c} R indices for data cut off at $I > 2\sigma(I)$. ^{d} wR2 = $\{\Sigma[w(F_o^2 - F_c^2)^2]/\Sigma[w(F_o^2)^2]\}^{1/2}$, where $w = 1/[\sigma^2(F_o^2) + (aP)^2 + bP]$, $P = (F_o^2 + 2F_c^2)/3$. ^{e} R indices for all data.

EPR Spectroscopy. Continuous wave X-band EPR spectra was recorded on a Bruker ELEXSYS E500 spectrometer. Spectra were simulated using the simulation package XSOPHE;[6] fluid solution spectra using the spin-Hamiltonian $\hat{H} = g\mu_B BS + aSI$, and frozen solution spectra using the spin-Hamiltonian $\hat{H} = \mu_B \mathbf{B}\cdot\mathbf{g}\cdot\mathbf{S} + \mathbf{S}\cdot\mathbf{A}\cdot\mathbf{I} + \mathbf{I}\cdot\mathbf{P}\cdot\mathbf{I} - \mu_n g_n \mathbf{B}\cdot\mathbf{I}$. The symbols have all their usual meanings.

Pulsed X-band EPR data were measured using a Bruker ELEXSYS E580 spectrometer equipped with an Oxford Instruments CF935 continuous Helium flow cryostat. Samples were prepared by dissolving **2** in the selected solvent mixtures (4:1 $CDCl_3/Cl_3CCN$, 4:1 CCl_4/Cl_3CCN, 4:1 CS_2/Cl_3CCN, 4:1 $Cl_3CCN/CDCl_3$) to a concentration of 1 mM and loading into 3.8 mm o.d. quartz EPR tubes. The solution samples were degassed via three freeze-pump-thaw cycles, followed by flame sealing. ESE-detected EPR spectra were collected at 10 K using a Hahn echo pulse sequence ($\pi/2 - \tau - \pi - \tau -$ echo) with a 4-step phase cycle, where $\pi/2 = 16$ ns, $\pi = 32$ ns and $\tau = 400$ ns. Simulations were performed as using XSOPHE[6] using the aforementioned spin-Hamiltonian. Phase memory times (T_M) were also measured with a Hahn echo pulse sequence. Decay curves were collected at field positions indicated on ESE spectra. Acquisition times were set to capture the top half of the spin echo and the acquired echo was integrated to obtain the spectrum. The data were phased by maximizing the sum of the data points in the real components of the spectrum and fit to the biexponential function $I(\tau) = y_0 + A_f \exp(-\tau/T_{M,f}) + A_s \exp(-\tau/T_{M,s})$, where f and s indicate fast and slow processes, respectively. Spin-lattice relaxation times (T_1) were collected at 10 K following the inversion recovery sequence ($\pi - T - \pi/2 - \tau - \pi - \tau -$ echo) with 4-step phase cycling in which $\pi/2 = 16$ ns, $\pi = 32$ ns, and T incremented from a starting value of 100 ns. The value of τ was selected to correspond to the maximum in the ESEEM at 400 ns. Acquisition times were set to capture the top half of the spin echo and the acquired echo was integrated to obtain the spectrum. The data were phased by maximizing the sum of the data points in the real components of the spectrum and fit to the biexponential function $I(\tau) = y_0 + A_f \exp(-\tau/T_{1,f}) + A_s \exp(-\tau/T_{1,s})$. Nutation measurements were performed at three different microwave powers with a nutation pulse of variable length (tipping) pulse followed by a Hahn echo sequence ($t_p - T - \pi/2 - \tau - \pi - \tau -$ echo). Data were collected employing 4-phase cycling, in which in which $\pi/2 = 16$ ns, $\pi = 32$ ns and $\tau = 400$ ns for nutation pulse lengths $T = 600$ ns. The tipping pulse, t_p, is augmented in 4 ns increments from a starting value of 4 ns. Nutation data was processed by subtracting a stretched exponential baseline from the echo decay, then zero-filling with 1024 or 2048 points, followed by a Fourier transform with a Hamming window.

Other Physical Methods. Cyclic voltammetry measurements were performed with a Metrohm Autolab P128 potentiostat. The electrode configuration consisted of a 2 mm glassy carbon working electrode, a platinum auxiliary electrode and a reference electrode consisting of $Ag/AgNO_3$ (0.01 M in MeCN) incorporated into a salt bridge containing supporting electrolyte (to minimize Ag^+ leakage). The

measurement were collected using a 1 mM solution of **1** dissolved in dichloromethane containing 0.1 M $[N(^{n}Bu)_4]PF_6$ as electrolyte. All reduction potentials are referenced versus the ferrocenium/ferrocene ($Fc^{+/0}$) couple. Electronic absorption spectra were recorded on a Shimadzu UVA 3600 spectrophotometer (range 200–1600 nm). Electrospray ionization (ESI) mass spectra were obtained on a Bruker micrOTOF-Q mass spectrometer. Elemental analysis were performed using an EA 1110 CHNS, CE-440 Elemental Analyzer.

Calculations. All calculations in this work were performed with the electronic structure program ORCA.[7] Geometry optimizations were carried out using the BP86 functional with dichloromethane as solvent.[8] A segmented all-electron relativistically contracted basis set of triple-ζ-quality (def2-TZVPP) was used for all atoms.[9] A scalar relativistic correction was applied using the zeroth-order regular approximation (ZORA) method[10] as implemented by van Wüllen.[11] In the context of ZORA, a one center approximation has been shown to introduce only minor errors to the final geometries. Auxiliary basis sets for all complexes used to expand the electron density in the calculations were chosen to match the orbital basis. The conductor like screening model (COSMO) was used for all calculations.[12] The self-consistent field calculations were tightly converged (1×10^{-8} E_h in energy, 1×10^{-7} E_h in the density change, and 1×10^{-7} in the maximum element of the DIIS[13] error vector). The geometry search for all complexes was carried out in redundant internal coordinates without imposing geometry constraints. The property calculations at the optimized geometries were done with the PBE0 hybrid functional[14] and the RIJCOSX algorithm to expedite calculation of the Hartree-Fock exchange.[15] In this case the same basis sets were used but with enhanced integration accuracy (SPECIALGRIDINTACC 10) for the metal and sulfur atoms. Calculation of spin-Hamiltonian parameters included a larger the integration grid (Grid5) and fully decontracted basis sets.[16] The use of all-electron calculations using scalar relativistic corrections mandated the effective nuclear charge (Z_{eff}) for Au be adjusted to 27.5, which corresponds to a one-electron spin-orbit coupling constant (ζ_{5d}) of ca. 4500 cm^{-1}. All other effective charges had their default values. Canonical orbitals and density plots were constructed using the program Molekel.[17]

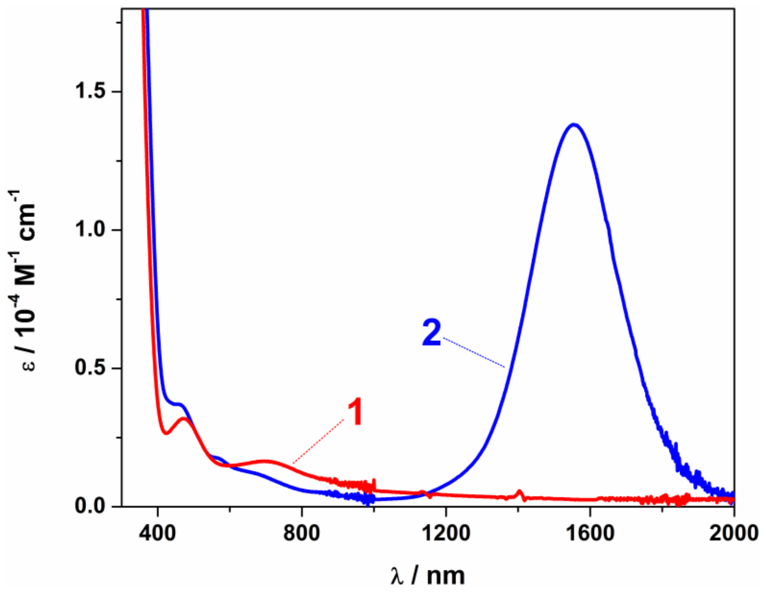

Fig. S1 Overlay of the electronic spectra of **1** and **2** recorded in CH_2Cl_2 at ambient temperature.

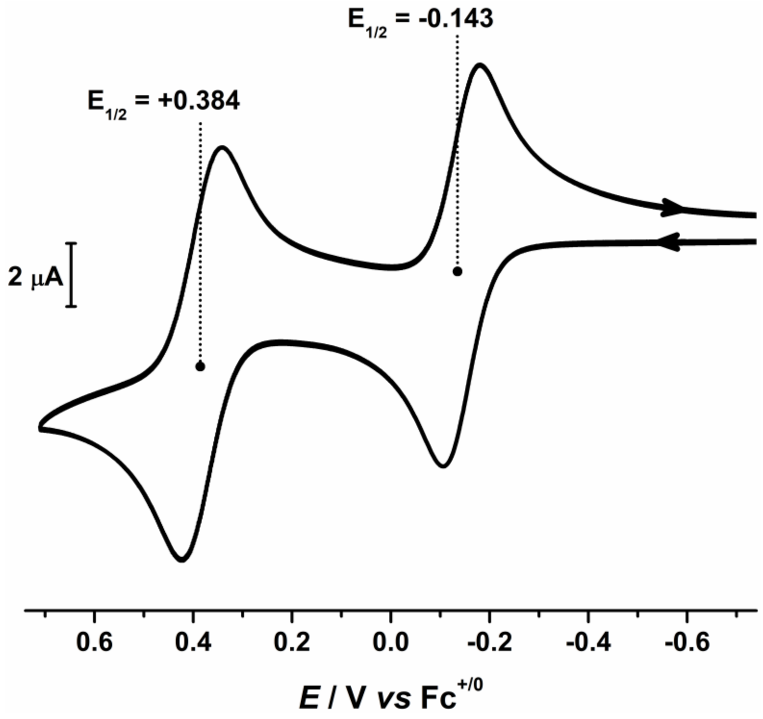

Fig. S2 Cyclic voltammogram of **1** in CH_2Cl_2 solution (0.10 M [N(^{n}Bu)$_4$]PF_6 supporting electrolyte) at 22 °C at a scan rate of 100 mV s^{-1}. Potentials are referenced versus the $Fc^{+/0}$ couple.

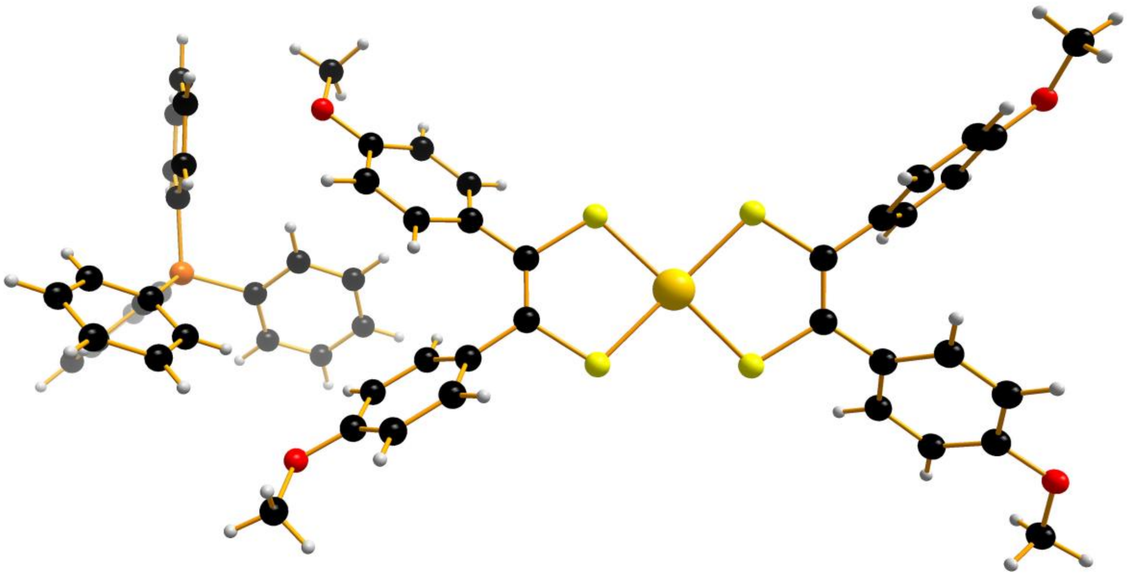

Fig. S3 Structure of the complex ion in crystals of **1** (color palette: Au, gold; S, canary; C, onyx; O, scarlet; P, pumpkin; H, alabaster).

Table S2 Geometry Optimized Coordinates for $[Au(adt)_2]^{1-}$

Au	1.96230628143174	6.90668020231104	9.11925912678939
S	2.80961994197524	4.73467133369890	8.81916046856897
S	2.90093814119653	7.46076887384144	7.03884328659729
S	1.08214596161679	6.38094858380935	11.23398618806152
S	1.04875749162892	9.05634171196861	9.37573434240507
O	6.42480913000891	0.02281887460201	5.98251015789347
O	5.79003002034103	6.53863728777067	1.25882153409500
O	-2.62172778305325	7.10584481428868	16.57922469643197
O	-1.87699598127055	14.02299191530276	12.54388646385502
C	3.63672666356426	4.81370275507454	7.24876757809949
C	3.65329121302678	5.94868966374995	6.49415482026685
C	0.23623896946782	7.86322652817795	11.72287780216986
C	0.24654238848881	8.99225796755220	10.95857578368775
C	4.33596428089161	3.55229656269780	6.90370992942186
C	3.69207877990072	2.30767764833393	7.01415745203739
H	2.64830354750774	2.27405157186524	7.33062282504546
C	4.34770671201865	1.10771931820271	6.71785927326769
H	3.80555627274459	0.16767431809476	6.81115405855798
C	5.68620058742089	1.13453604963550	6.30137141259437
C	6.35338703598308	2.36769026207593	6.19747879940502
H	7.39967227560710	2.37820483636589	5.88780612478795
C	5.68861122478782	3.55042871482610	6.49891666634504
H	6.22584113445848	4.49698431213613	6.42929424083658
C	5.78140291766010	-1.25462867082015	6.09406517121407
H	5.45472780935763	-1.44717188079765	7.12782089513578
H	6.53670300733910	-1.99311491739990	5.80414171840377
H	4.91631982438012	-1.32576859294039	5.41606880729882
C	4.21967754651239	6.06096618120504	5.12746737091767
C	3.86445111896564	5.15807974168810	4.11140678713079
H	3.17150596502019	4.34663376442813	4.33849428227355
C	4.36802802028445	5.27487441829067	2.81223941216694
H	4.05995056280742	4.55521655450767	2.05502121456540
C	5.24499317278827	6.32443304305024	2.49932877511856
C	5.60631702964308	7.24420945500048	3.49850003700770
H	6.29055869952175	8.05694103594064	3.24767454409172
C	5.09742298974551	7.11283537269731	4.78627744637938
H	5.39058489513822	7.83283276659660	5.55245567166871
C	5.44331477502156	5.61864277824516	0.21410710034101
H	5.76915943646652	4.59586481189802	0.45945153229734
H	5.97376720232328	5.96944733741992	-0.67742016276189
H	4.35862981647810	5.62310324033071	0.02296064191807
C	-0.52212148477581	7.69817719748636	12.98671145424498
C	0.07997625539257	7.14572834743199	14.13787182188657
H	1.13391072251799	6.86478190009044	14.10703719935259
C	-0.63839698246360	6.96239517730261	15.31487173938349
H	-0.16071084732528	6.54162142460540	16.20151430551441
C	-1.99487250022010	7.32284407820898	15.37799096990246
C	-2.61835223249577	7.86203990090076	14.24307495985733
H	-3.67203701234048	8.13717550066132	14.25760051831534
C	-1.88320349719478	8.03766642240442	13.06770735440234
H	-2.38268295731277	8.44492018243625	12.18773516444392
C	-4.00950219740208	7.45630154977373	16.67382699229269
H	-4.16260797119323	8.53144460237974	16.48843184719814
H	-4.30694073032304	7.21468976692222	17.69995076953808
H	-4.61725237474185	6.87094441502766	15.96664423352042
C	-0.33634571093276	10.29647821910879	11.35658106535664
C	-0.06052378606827	10.86923635584578	12.61683071956341
H	0.58421507588808	10.33456988284355	13.31529613086873
C	-0.58503008001732	12.10244486596845	12.98378462647859
H	-0.36139967266469	12.53591548051826	13.96003362244383
C	-1.40340965189933	12.81734073386701	12.09163140317644
C	-1.67759125982366	12.27856756132221	10.82690520833624

H	-2.30510651990233	12.81050861650009	10.11287272526958
C	-1.14376524536884	11.03479968843097	10.47412062845994
H	-1.37166141112721	10.62391916282348	9.48915273044537
C	-2.70293739977026	14.78670416488542	11.65341200279077
H	-2.15429671926780	15.06299800183196	10.73938573777526
H	-2.97533705961950	15.69259907274076	12.20520548653527
H	-3.61486882874491	14.23247118195872	11.38131930625873

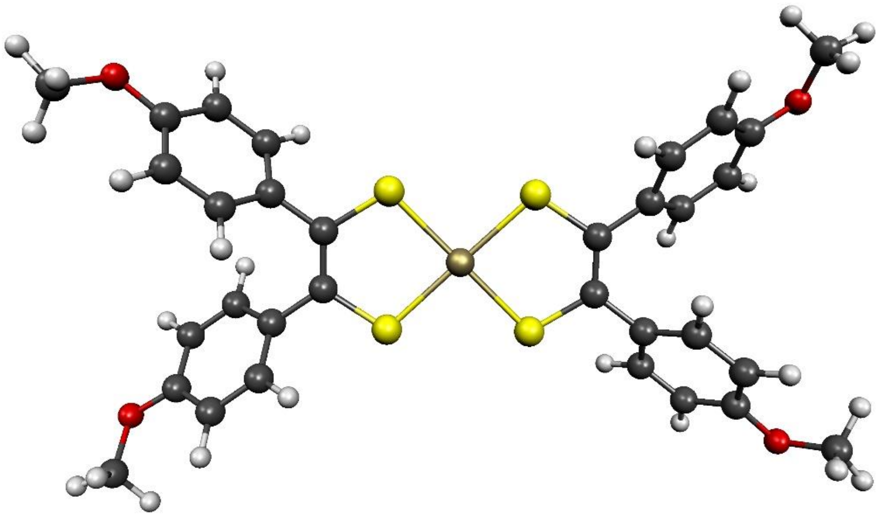

Fig. S4 Geometry optimized structure of $[Au(adt)_2]^{1-}$

Table S3 Geometry Optimized Coordinates for **2**

Au	1.93713316707976	6.89705687473567	9.10773435821241
S	2.81589645830381	4.75163650463472	8.81921906830205
S	2.84426962340421	7.44489118441999	7.02865963969128
S	1.02990299364328	6.35938120813379	11.19148597972429
S	1.04832718081823	9.03952522837965	9.37961471097849
O	6.45929395114957	0.08336239538893	6.02210786539775
O	5.71027076739101	6.54737722140889	1.26099143832105
O	-2.65364792904097	7.06098277518437	16.51927322891990
O	-1.79240720891744	14.00484930259426	12.56042805998715
C	3.61503191270193	4.82307395331260	7.26617779286605
C	3.60802527311951	5.96874393580900	6.49134163340266
C	0.21668603478611	7.82178311828736	11.69352713442752
C	0.24769043802603	8.97602629414517	10.93111809432008
C	4.32785834599251	3.57545568616660	6.91807901864157
C	3.70172872042996	2.32323263770724	7.05349594059224
H	2.65985199152067	2.27664871900785	7.37404579793798
C	4.37317796782217	1.13355502587265	6.76351450381532
H	3.84746237098576	0.18582056787614	6.86812132003677
C	5.70880777846157	1.17935569482398	6.33446470645058
C	6.35560729339900	2.42358584371369	6.20617706954454
H	7.39822029498107	2.44600676249628	5.88659339788315
C	5.67515161147602	3.59782884129569	6.49273423312832
H	6.19618607672273	4.55150696685434	6.40479579326153
C	5.84632052264239	-1.21037165335126	6.15619262232020
H	5.53939529758638	-1.39612408101240	7.19645005763733
H	6.61641823406041	-1.93186388162207	5.86424667353019
H	4.97628107783719	-1.30650080433091	5.48900474905229
C	4.17212563861515	6.07331227610051	5.12804093430384
C	3.85796089804534	5.12996846763068	4.13344200558988
H	3.20175204069470	4.29287582077393	4.37396259661765
C	4.34903890615525	5.24995718433414	2.83302018162909
H	4.07216687487890	4.50465873924672	2.08904270765945
C	5.17807489778980	6.33429225623683	2.49898615198702
C	5.50041260998661	7.28904830090245	3.48048681765824
H	6.14979741843123	8.12421588350603	3.21357883323387
C	4.99942595293849	7.16077237777564	4.76917220481634
H	5.26645998976676	7.90388843121483	5.52245427591749
C	5.41622809247865	5.59014098198828	0.22926272270116
H	5.79189960078928	4.59149725015355	0.49819142589355
H	5.93581632174786	5.95211620851901	-0.66362109938027
H	4.33445990073715	5.54000773549123	0.03305893561848
C	-0.54228852880584	7.66582303831192	12.95275934552787
C	0.04546785694105	7.06529762827379	14.08809833481386
H	1.09133690550971	6.75700465626866	14.05265461510544
C	-0.67771737217071	6.88099304577312	15.25916300553952
H	-0.21399575221015	6.42924683740823	16.13734719399746
C	-2.02366465273852	7.28318748439857	15.32933290264510
C	-2.63066736779843	7.87029188705891	14.20669650114792
H	-3.67520326027574	8.17631665459944	14.22840787271630
C	-1.89100970161520	8.05561297079404	13.03917273101530
H	-2.37779491723298	8.49882170144343	12.17002051903382
C	-4.03113249536627	7.45640310389457	16.63255835072885
H	-4.14596451450051	8.53998267490578	16.47463286445433
H	-4.32738437056486	7.19925187996097	17.65468503683947
H	-4.66009330957020	6.90717053906600	15.91608628974124
C	-0.32139734024845	10.27608874477957	11.34572723667556
C	-0.04569569008409	10.82165291240960	12.61919892043184
H	0.58304038863354	10.26795908581368	13.31681381730283
C	-0.54394734194331	12.06114160679367	12.99148263170436
H	-0.31897109288978	12.48101913891627	13.97280583294548
C	-1.34219671091109	12.80239211869922	12.09909455917081
C	-1.62153828254909	12.28275415237384	10.82580547097852

H	-2.23857281856990	12.83227128461887	10.11660954924328
C	-1.10822441434281	11.03633826232750	10.46181758253155
H	-1.34311492665305	10.63730505881374	9.47385879646584
C	-2.59975053543720	14.80289366784942	11.67792725590617
H	-2.03896149995395	15.08118425112267	10.77277266830762
H	-2.85305147189720	15.70388794067407	12.24566257306134
H	-3.52139917219296	14.27165343684446	11.39586895933974

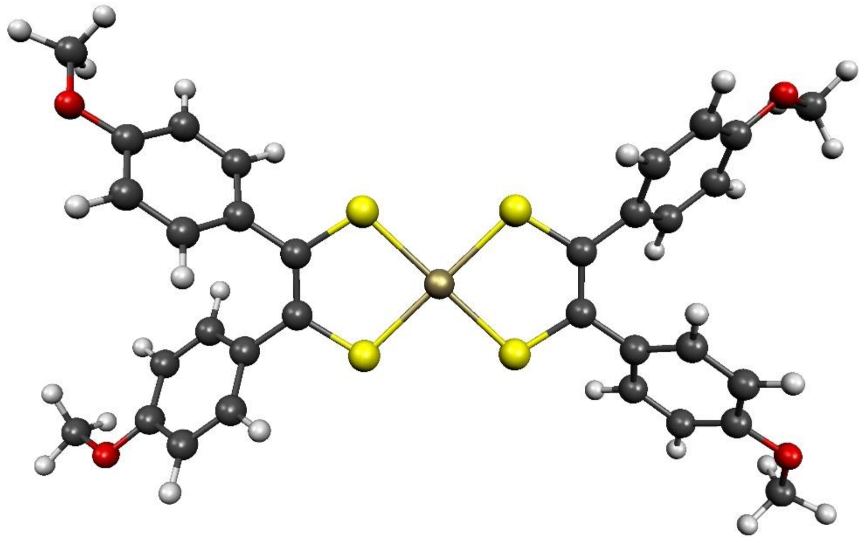

Fig. S5 Geometry optimized structure of **2**

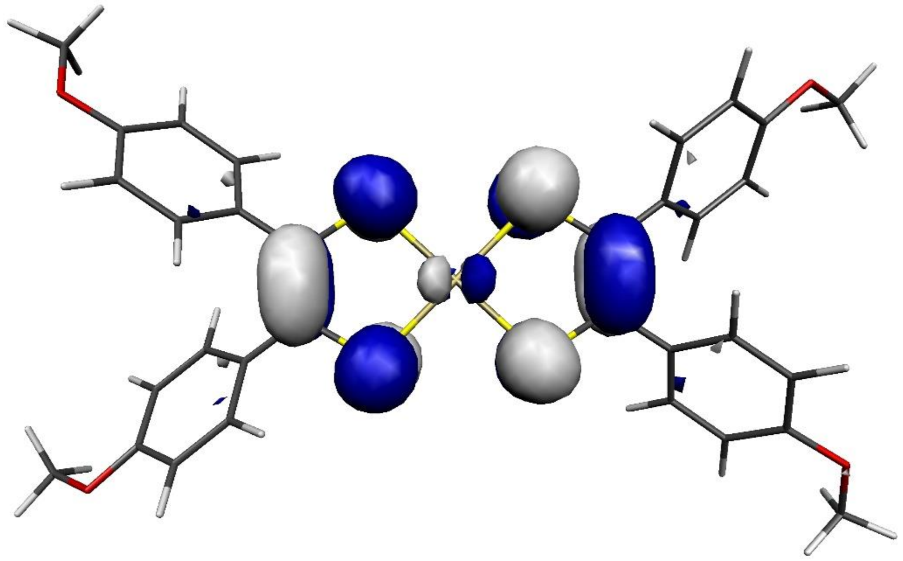

Fig. S6 Depiction of the b_{2g} magnetic orbital in **2**

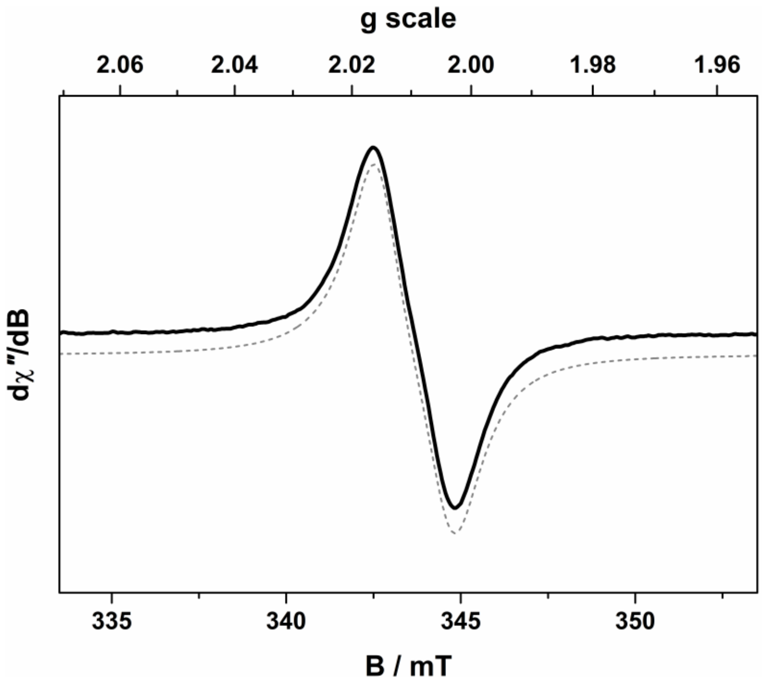

Fig. S7 X-band EPR spectrum of **2** recorded in CH_2Cl_2 solution at 293 K (experimental conditions: frequency, 9.6657 GHz; power, 6.3 mW; modulation, 0.5 mT). Experimental data are represented by the black line; simulation is depicted by the dashed trace.

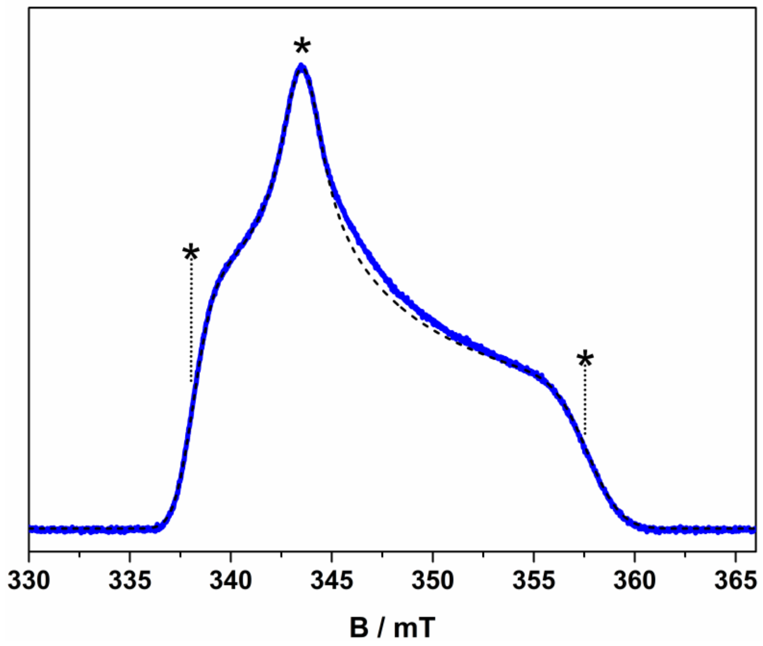

Fig. S8 ESE detected EPR spectrum (blue line) and simulation (dashed line) of a 1 mM solution of **2** in 4:1 CCl_4/Cl_3CCN recorded at 10 K. Asterisks indicates field positions for relaxation measurements.

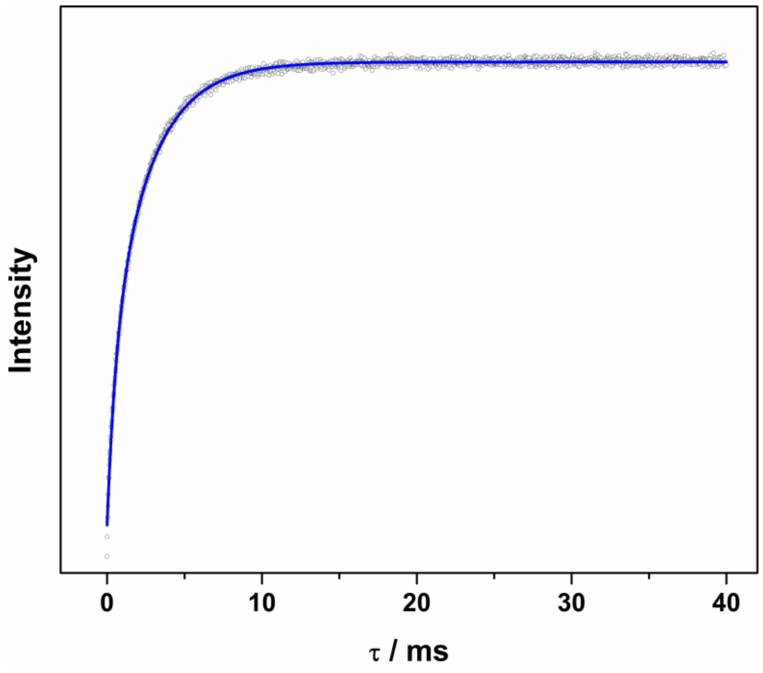

Fig. S9 Inversion recovery data (open circles) and biexponential fit (blue line) of a 1 mM solution of **2** in 4:1 CCl_4/Cl_3CCN recorded at 10 K and 343.5 mT.

Table S4 Fit parameters for inversion recovery data of **2** in 4:1 CCl_4/Cl_3CCN at 343.5 mT

T	A_f	$T_{1,f}$ / µs	A_s	$T_{1,s}$ / µs
5	0.385(3)	7000(100)	0.414(2)	58300(400)
10	0.72(1)	560(10)	1.41(1)	2630(20)
20	2.54(6)	44.7(9)	5.51(6)	139.9(8)
40			6.23(2)	9.65(3)
60			6.0(4)	3.1(7)
80			27.2(1)	1.61(1)

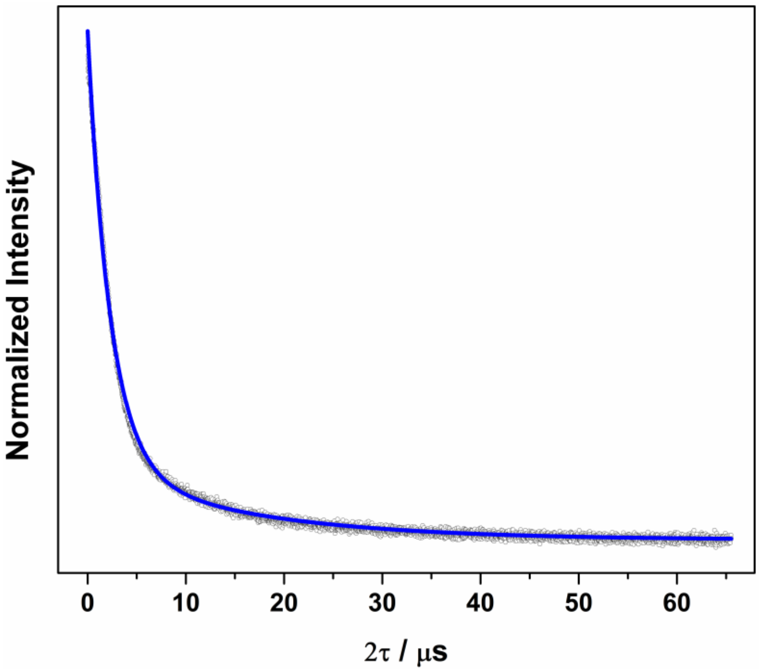

Fig. S10 Hahn-echo decay curve (open circles) and biexponential fit (blue line) of a 1 mM solution of **2** in 4:1 CCl_4/Cl_3CCN recorded at 10 K and 343.5 mT.

Table S5 Biexponental fit parameters for Hahn-echo decay curves of **2** in 4:1 CCl_4/Cl_3CCN at 343.5 mT

T	A_f	$T_{M,f}$ / µs	A_s	$T_{M,s}$ / µs
5	29.72(5)	0.194(1)	3.75(3)	11.5(1)
10	55.7(1)	2.36(1)	12.0(1)	15.7(2)
20	80.3(3)	2.20(1)	15.7(3)	13.7(1)
40	101(2)	1.70(2)	13(2)	8.0(6)
60	145.6(2)	1.13(1)	30.2(1)	2.52(2)
80	220(10)	0.29(3)	48(8)	2.6(5)

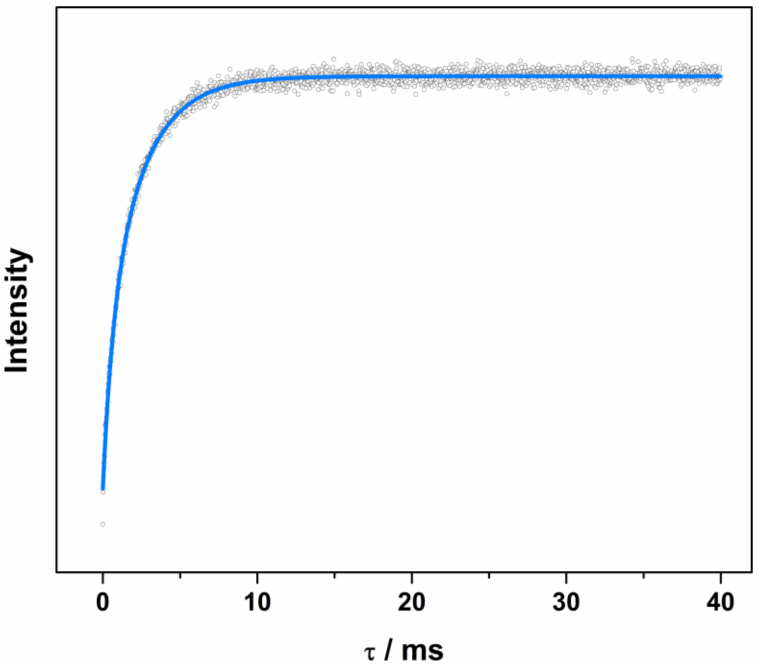

Fig. S11 Inversion recovery data (open circles) and biexponential fit (blue line) of a 1 mM solution of **2** in 4:1 CCl_4/Cl_3CCN recorded at 10 K and 338.1 mT.

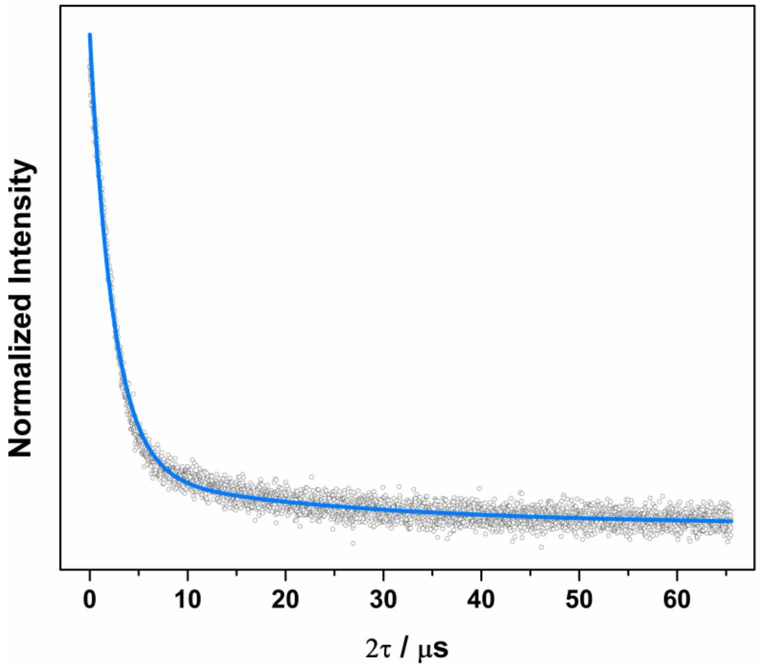

Fig. S12 Hahn-echo decay curve (open circles) and biexponential fit (blue line) of a 1 mM solution of **2** in 4:1 CCl_4/Cl_3CCN recorded at 10 K and 338.1 mT.

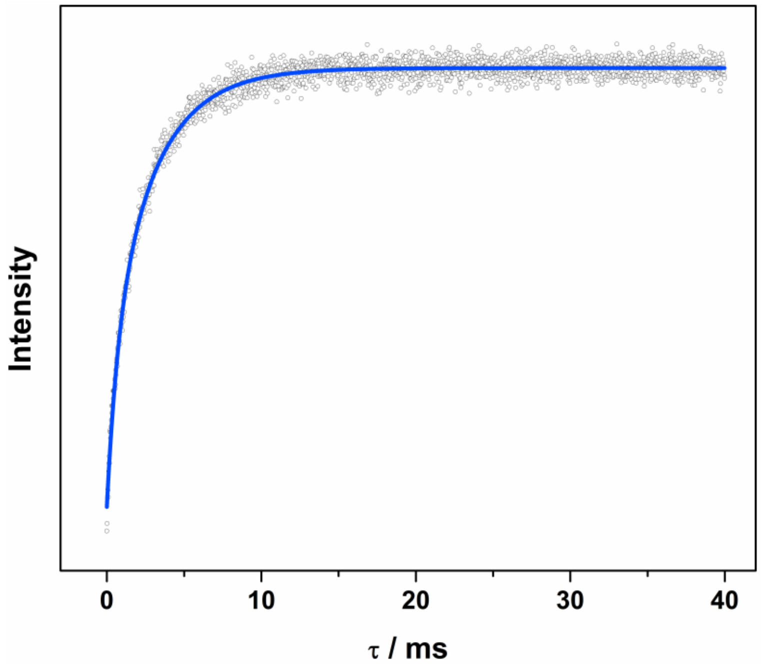

Fig. S13 Inversion recovery data (open circles) and biexponential fit (blue line) of a 1 mM solution of **2** in 4:1 CCl_4/Cl_3CCN recorded at 10 K and 357.5 mT.

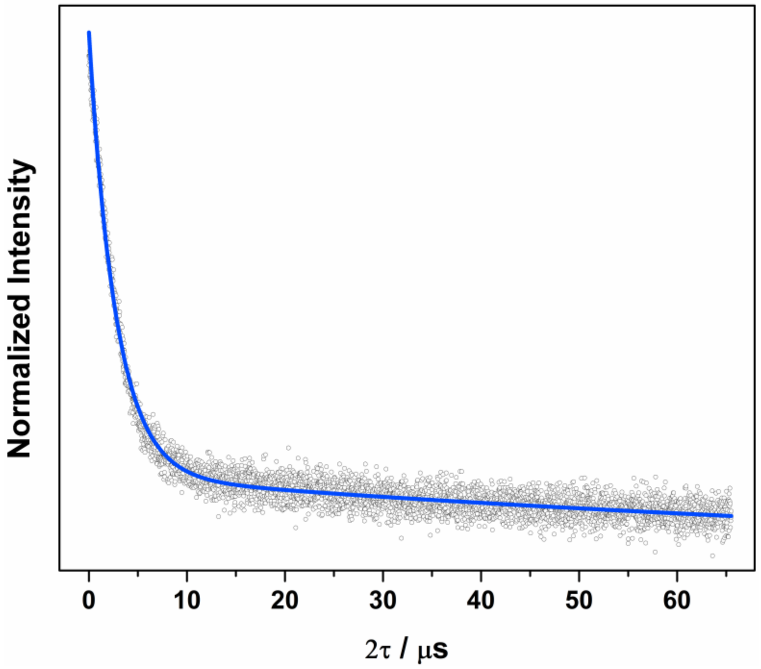

Fig. S14 Hahn-echo decay curve (open circles) and biexponential fit (blue line) of a 1 mM solution of **2** in 4:1 CCl_4/Cl_3CCN recorded at 10 K and 357.5 mT.

Table S6 Biexponental fit parameters for inversion recovery data of **2** in 4:1 CCl_4/Cl_3CCN at 10 K

**B / mT**	A_f	$T_{1,f}$ / ms	A_s	$T_{1,s}$ / ms
338.1	0.97(5)	0.62(4)	1.95(5)	2.43(4)
343.5	0.72(1)	0.56(1)	1.41(1)	2.63(2)
357.5	1.50(8)	0.70(5)	3.08(9)	2.95(6)

Table S7 Biexponental fit parameters for Hahn-echo decay curves of **2** in 4:1 CCl_4/Cl_3CCN at 10 K

**B / mT**	A_f	$T_{M,f}$ / μs	A_s	$T_{M,s}$ / μs
338.1	94.0(4)	2.3(1)	18.8(2)	15.8(1)
343.5	55.7(1)	2.36(1)	12.0(1)	15.7(2)
357.5	120.7(5)	2.50(2)	17.6(4)	17.6(1)

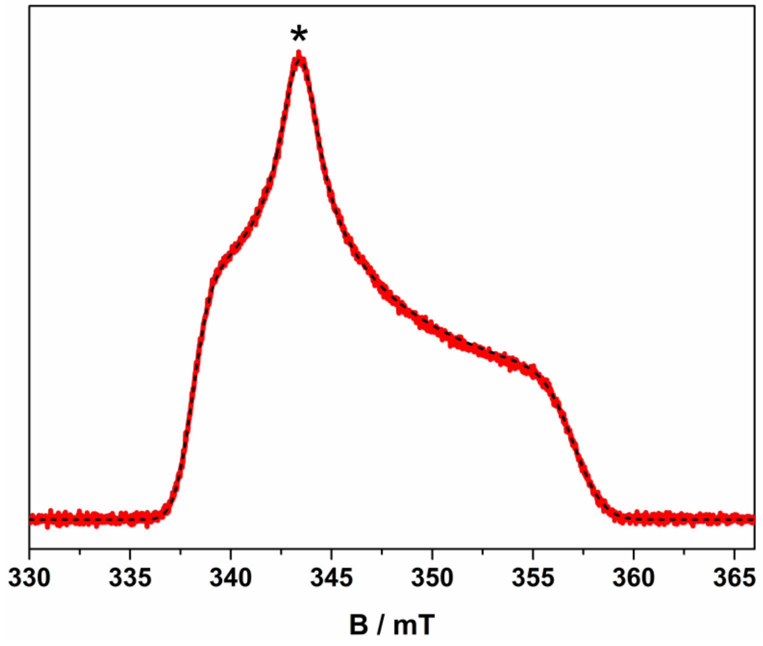

Fig. S15 ESE detected EPR spectrum (red line) and simulation (dashed line) of a 1 mM solution of **2** in 4:1 $CDCl_3/Cl_3CCN$ recorded at 10 K. Asterisk indicates field position for relaxation measurements.

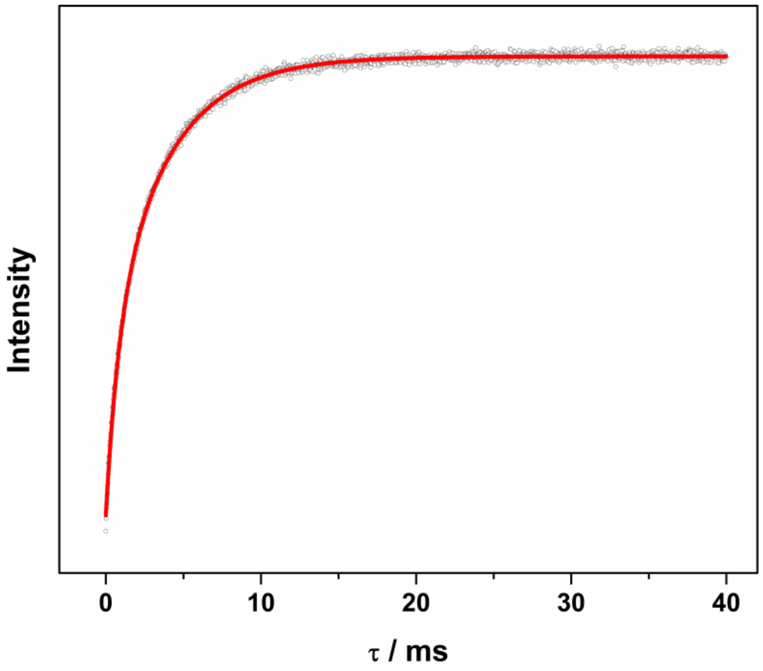

Fig. S16 Inversion recovery data (open circles) and biexponential fit (red line) of a 1 mM solution of **2** in 4:1 $CDCl_3/Cl_3CCN$ recorded at 10 K and 343.5 mT.

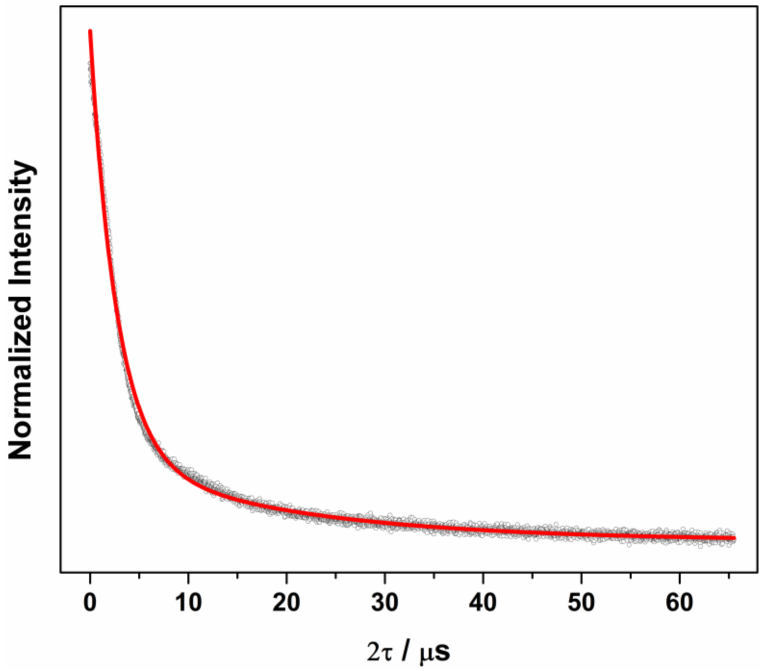

Fig. S17 Hahn-echo decay curve (open circles) and biexponential fit (red line) of a 1 mM solution of **2** in 4:1 CCl_4/Cl_3CCN recorded at 10 K and 343.5 mT.

Table S8 Biexponential fit parameters for inversion recovery data of **2** in 4:1 $CDCl_3$/Cl_3CCN at 343.5 mT

T	A_f	$T_{1,f}$ / ms	A_s	$T_{1,s}$ / ms
5	0.491(4)	17.2(2)	0.727(4)	85.8(5)
10	0.475(6)	0.83(2)	0.820(7)	3.81(2)
20	1.20(2)	0.044(1)	2.71(2)	0.183(1)

Table S9 Biexponental fit parameters for Hahn-echo decay curves of **2** in 4:1 $CDCl_3$/Cl_3CCN at 343.5 mT

T	A_f	$T_{M,f}$ / μs	A_s	$T_{M,s}$ / μs
5	41.1(2)	2.58(2)	6.7(1)	17.0(5)
10	46.0(1)	2.65(1)	6.7(1)	19.3(5)
20	102.9(4)	2.55(2)	14.6(4)	13.8(4)

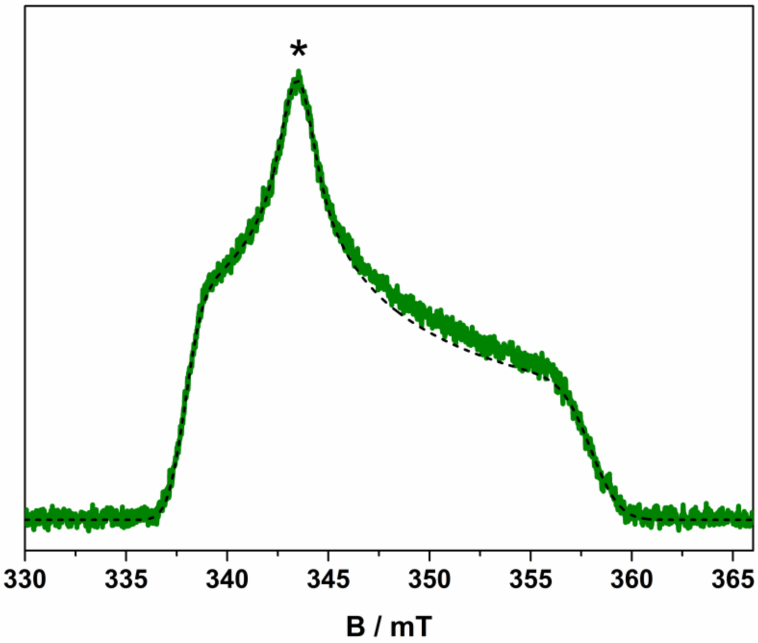

Fig. S18 ESE detected EPR spectrum (green line) and simulation (dashed line) of a 1 mM solution of **2** in 4:1 CS_2/CCl_4 recorded at 10 K. Asterisk indicates field position for relaxation measurements.

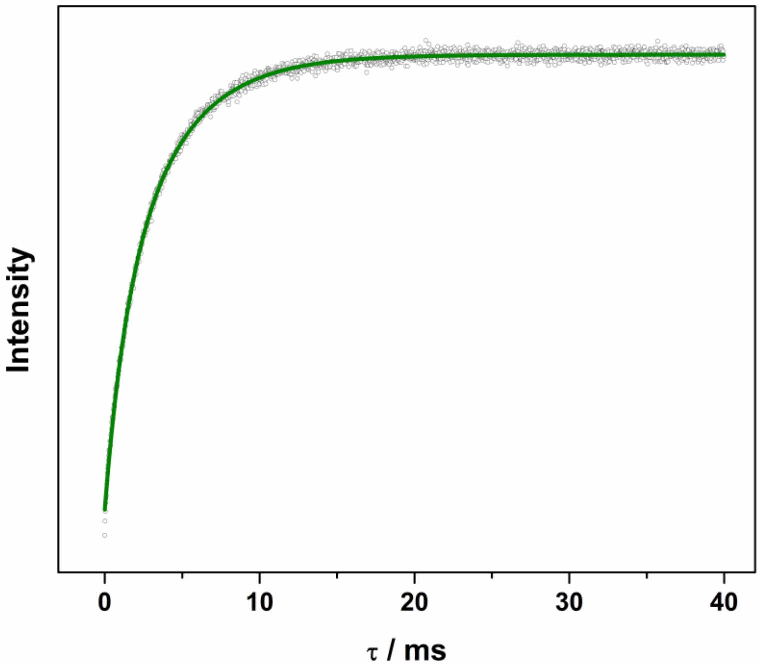

Fig. S19 Inversion recovery data (open circles) and biexponential fit (green line) of a 1 mM solution of **2** in 4:1 CS_2/CCl_4 recorded at 10 K and 343.5 mT.

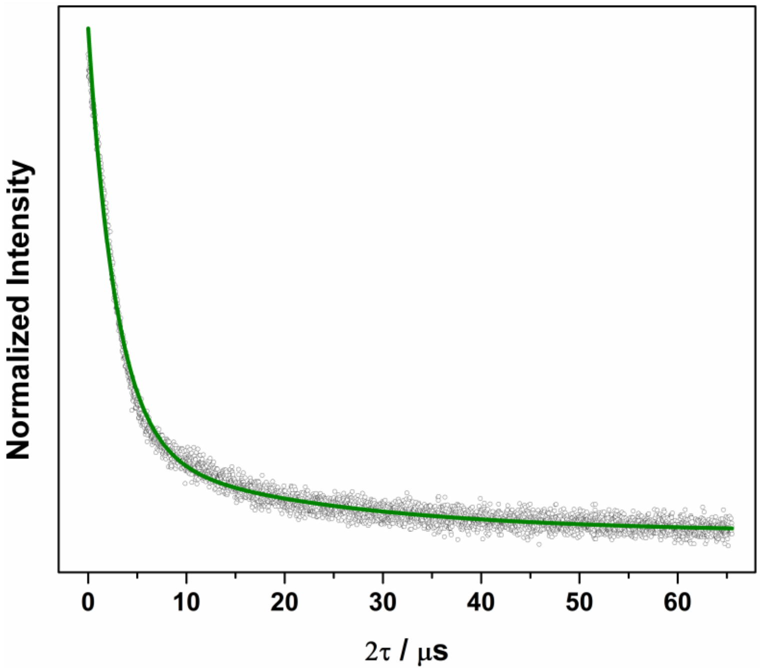

Fig. S20 Hahn-echo decay curve (open circles) and biexponential fit (green line) of a 1 mM solution of **2** in 4:1 CS_2/CCl_4 recorded at 10 K and 343.5 mT.

Table S10 Biexponential fit parameters for inversion recovery data of **2** in 4:1 CS_2/CCl_4 at 343.5 mT

T	A_f	$T_{1,f}$ / ms	A_s	$T_{1,s}$ / ms
5	0.346(8)	15.1(6)	0.766(7)	92.0(8)
10	3.8(2)	1.31(5)	7.5(2)	3.89(5)
20	4(1)	0.11(1)	4(1)	0.19(2)

Table S11 Biexponental fit parameters for Hahn-echo decay curves of **2** in 4:1 CS_2/CCl_4 at 343.5 mT

T	A_f	$T_{M,f}$ / μs	A_s	$T_{M,s}$ / μs
5	138(1)	2.84(4)	23(1)	16.9(8)
10	143.7(7)	2.79(2)	22.8(6)	21.0(7)
20	115(1)	2.69(4)	18(1)	17.1(4)

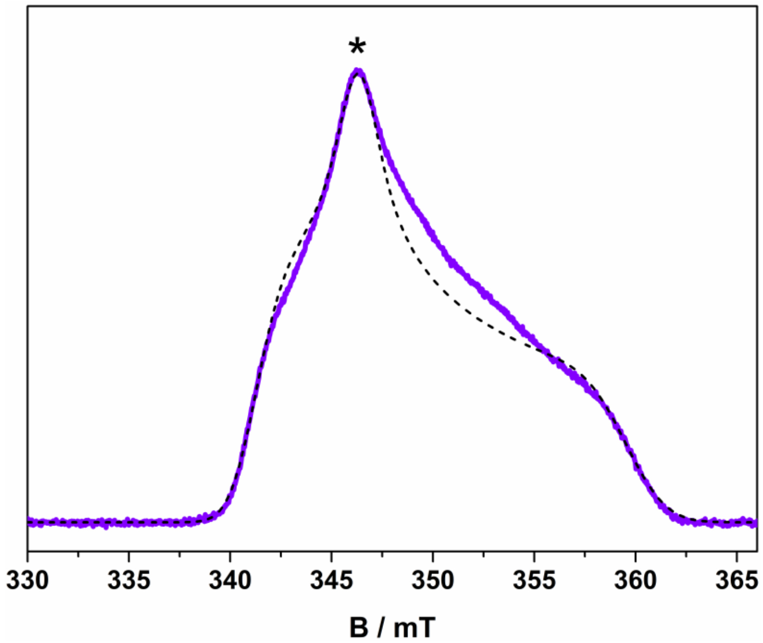

Fig. S21 ESE detected EPR spectrum (violet line) and simulation (dashed line) of a 1 mM solution of **2** in 4:1 Cl_3CCN/CCl_4 recorded at 10 K. Asterisk indicates field position for relaxation measurements.

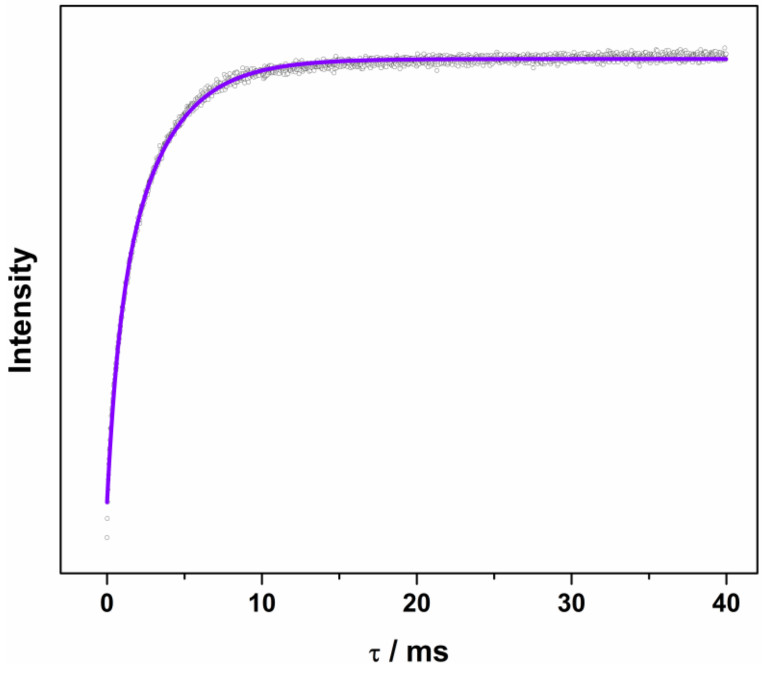

Fig. S22 Inversion recovery data (open circles) and biexponential fit (violet line) of a 1 mM solution of **2** in 4:1 Cl_3CCN/CCl_4 recorded at 10 K and 346.2 mT.

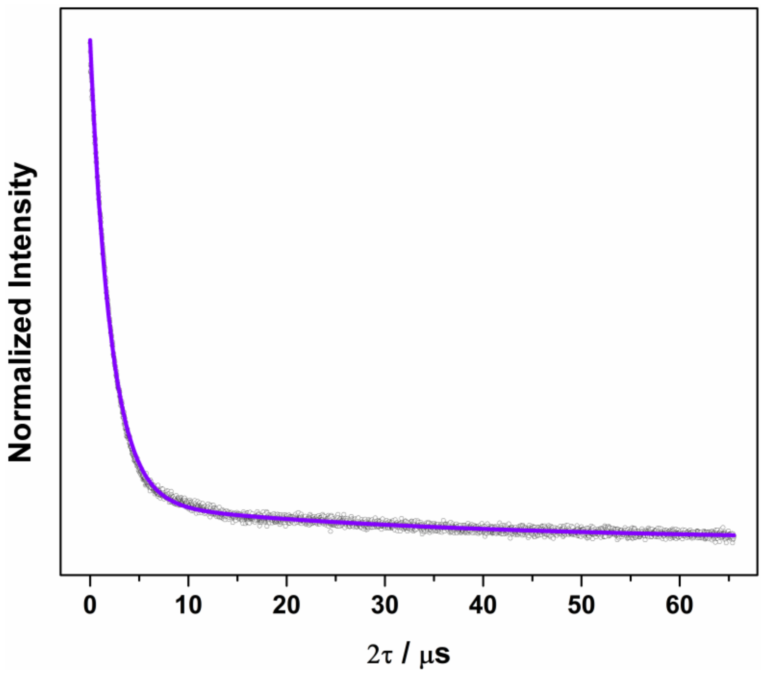

Fig. S23 Hahn-echo decay curve (open circles) and biexponential fit (violet line) of a 1 mM solution of **2** in 4:1 Cl_3CCN/CCl_4 recorded at 10 K and 346.2 mT.

Table S12 Biexponential fit parameters for inversion recovery data of **2** in 4:1 Cl_3CCN/CCl_4 at 346.2 mT

T	A_f	$T_{1,f}$ / ms	A_s	$T_{1,s}$ / ms
5	0.497(3)	7.97(9)	0.441(3)	56.1(3)
10	0.362(7)	0.7(2)	0.709(8)	3.09(2)
20	0.56(1)	0.023(1)	2.78(1)	0.122(1)

Table S13 Biexponental fit parameters for Hahn-echo decay curves of **2** in 4:1 Cl_3CCN/CCl_4 at 346.2 mT

T	A_f	$T_{M,f}$ / μs	A_s	$T_{M,s}$ / μs
5	35.6(1)	1.85(1)	2.59(6)	14.0(4)
10	40.1(1)	1.95(5)	2.95(3)	15.2(6)
20	64.4(1)	1.8(1)	6.23(8)	14.4(4)

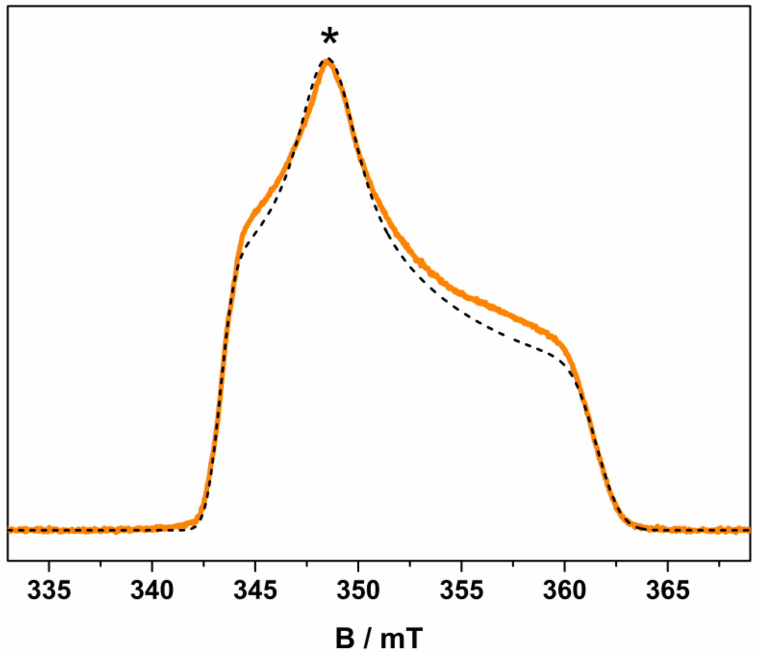

Fig. S24 ESE detected EPR spectrum (orange line) and simulation (dashed line) of a polycrystalline sample of **2** diluted 2% in [Ni(adt)$_2$] recorded at 10 K. Asterisk indicates field position for relaxation measurements.

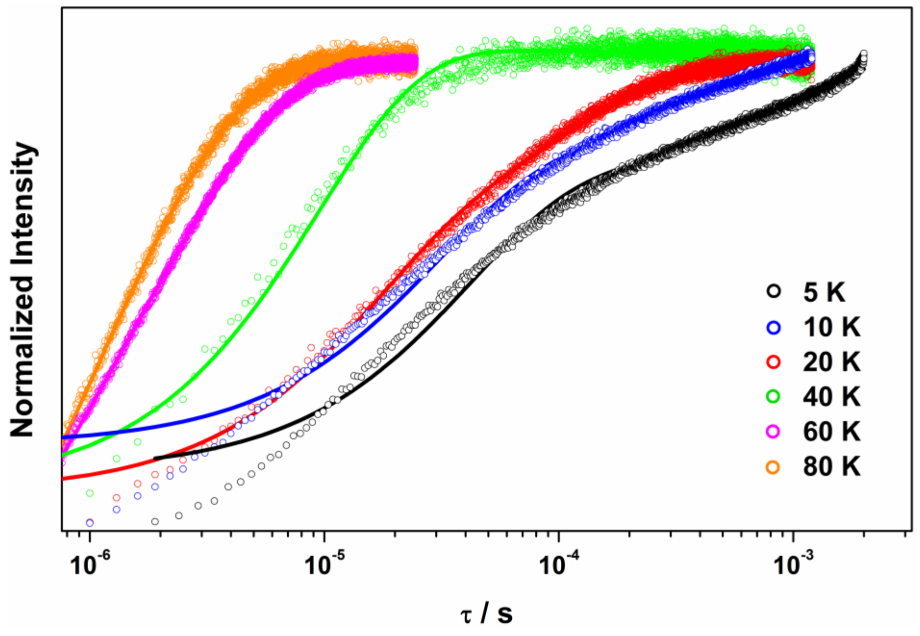

Fig. S25 Inversion recovery data (open circles) and biexponential fit (solid line) of a polycrystalline sample of **2** diluted 2% in [Ni(adt)$_2$] at 348.5 mT.

Table S14 Biexponential fit parameters for inversion recovery data of **2** diluted 2% in $[Ni(adt)_2]$ at 348.5 mT

T	A_f	$T_{1,f}$ / µs	A_s	$T_{1,s}$ / µs
5	3.14(1)	41.5(3)	1.56(1)	1030(10)
10	3.33(1)	30.2(2)	1.75(1)	336(3)
20	2.81(2)	18.5(3)	1.91(2)	123(1)
40	4.9(4)	5.0(5)	3.1(5)	16(1)
60	31.6(6)	1.05(3)	49.0(7)	3.08(2)
80	17.9(7)	0.72(4)	27(1)	2.04(2)

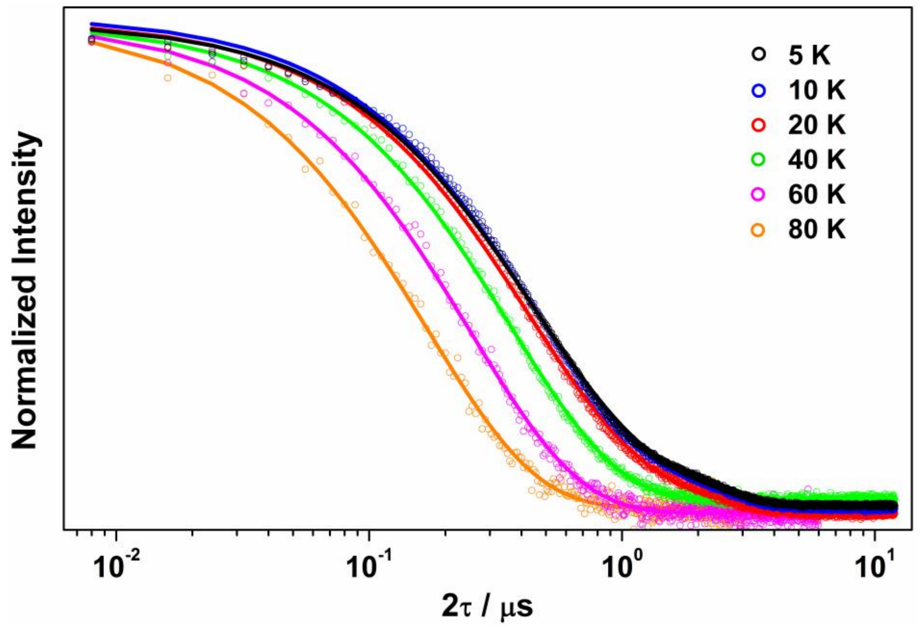

Fig. S26 Hahn-echo decay curve (open circles) and biexponential fit (solid line) of a polycrystalline sample of **2** diluted 2% in $[Ni(adt)_2]$ at 348.5 mT.

Table S15 Fit parameters for Hahn-echo decay curves of **2** diluted 2% in $[Ni(adt)_2]$ at 348.5 mT

T	A_f	$T_{M,f}$ / µs	A_s	$T_{M,s}$ / µs
5	134.0(9)	0.406(3)	25.6(9)	1.36(3)
10	88.6(7)	0.409(3)	14.3(7)	1.44(4)
20	92.9(9)	0.389(4)	13.4(9)	1.33(5)
40			40.7(9)	0.36(1)
60			71(2)	0.25(1)
80			667(5)	0.17(1)

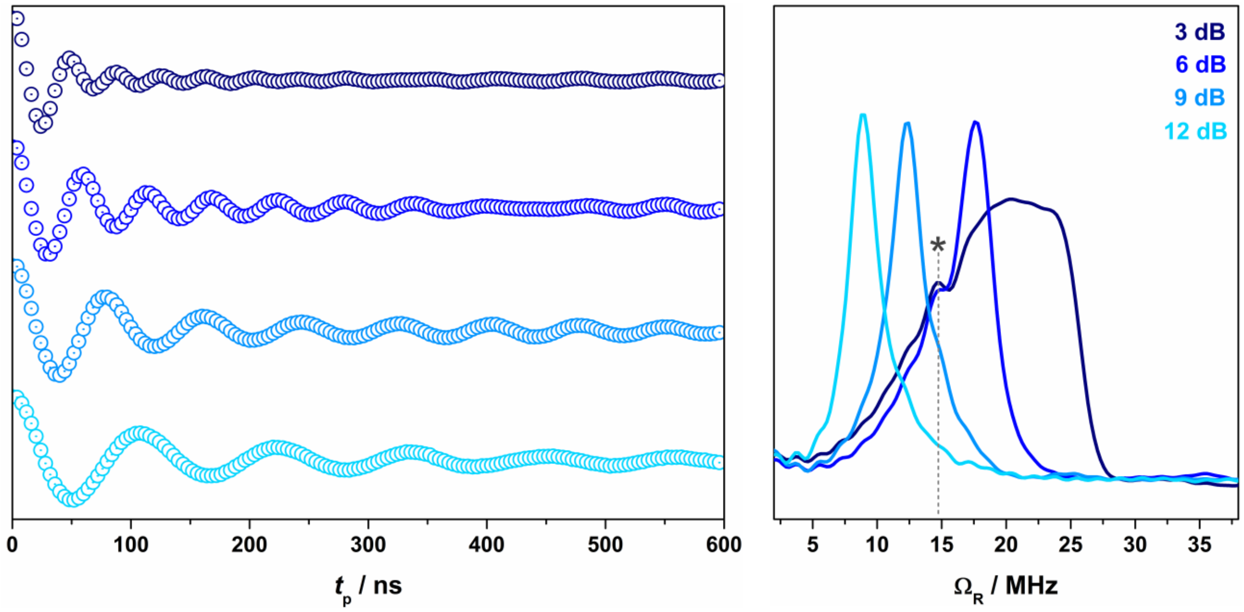

Fig. S27 Rabi oscillations (left) and corresponding frequencies from the Fourier transfer of the data (right) for **2** in 4:1 CCl_4/Cl_3CCN at 10 K and 343.5 mT from variable power nutation measurements. The asterisk in the Fourier transform data indicate the peak matching the Larmor frequency of ^1H (14.6 MHz) within error.[18]

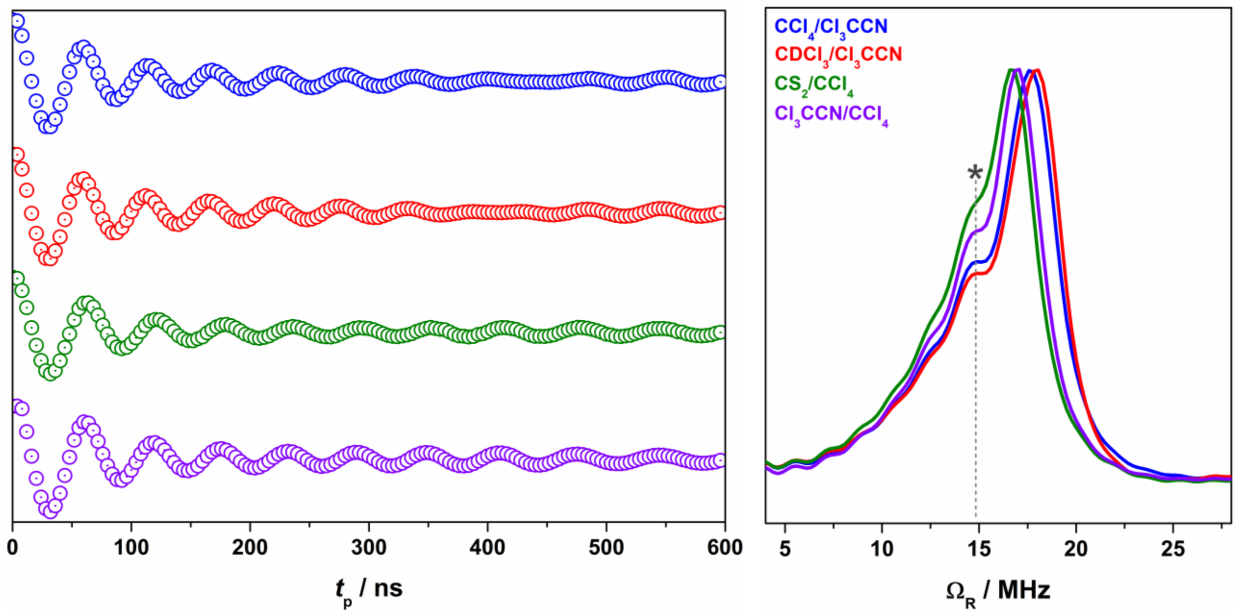

Fig. S28 Nutation data (left) and Fourier transforms of the data (right) for **2** in 4:1 CCl_4/Cl_3CCN (blue), 4:1 $CDCl_3/Cl_3CCN$ (red), 4:1 CS_2/CCl_4 (green), and 4:1 Cl_3CCN/CCl_4 (violet) at 10 K and 343.5 mT. The asterisk in the Fourier transform data indicate the peak matching the Larmor frequency of ^1H (14.6 MHz) within error.[18]

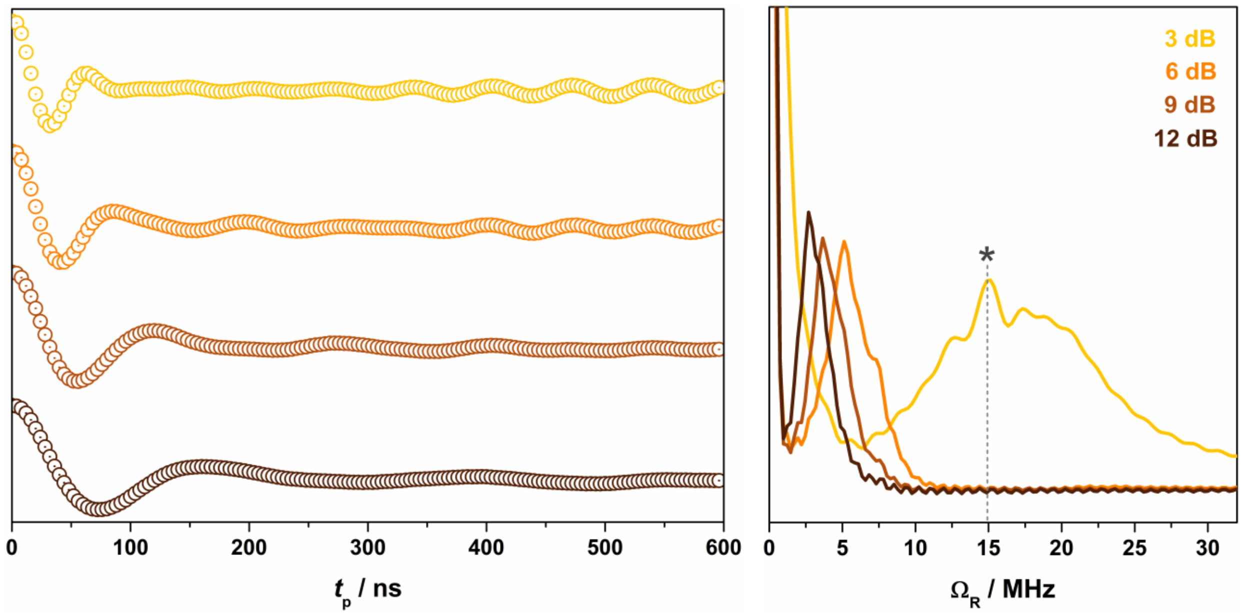

Fig. S29 Rabi oscillations (left) and corresponding frequencies from the Fourier transfer of the data (right) for **2** in diluted 2% in [Ni(adt)$_2$] at 10 K and 348.5 mT from variable power nutation measurements. The Rabi frequency at 3 dB microwave power is obscured in the Fourier transform from coupling to the Larmor frequency of ^{1}H (14.6 MHz) within error.[18]

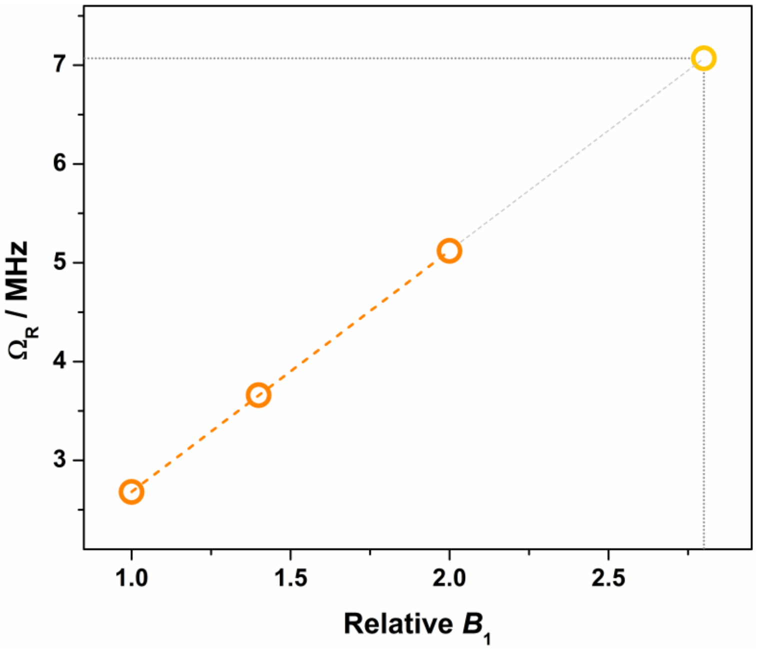

Fig. S30 Linear dependence of the oscillation frequency (Ω_R) with respect to the B_1 field for polycrystalline **2** diluted 2% in [Ni(adt)$_2$]. Pumpkin-colored open circles indicate oscillation frequency and the corresponding dashed line the line of best fit. The gray extrapolation line is used to estimate the Rabi frequency for relative B_1 = 2.82 (microwave power = 3 dB) of 7.07 MHz as indicated by sight lines.

References

1. P. Chandrasekaran, A. F. Greene, K. Lillich, S. Capone, J. T. Mague, S. DeBeer and J. P. Donahue, *Inorg. Chem.*, 2014, **54**, 9192.
2. G. M. Sheldrick, *Acta Crystallogr. Sect. A*, 1990, **46**, 467.
3. G. M. Sheldrick, *Acta Crystallogr. Sect. A*, 2008, **64**, 112.
4. L. J. Farrugia, *J. Appl. Cryst.*, 1999, **32**, 837.
5. R. C. Clark and J. S. Reid, *Acta Crystallogr. Sect. A*, 1995, **51**, 887.
6. G. R. Hanson, K. E. Gates, C. J. Noble, M. Griffin, A. Mitchell and S. Benson, *J. Inorg. Biochem.*, 2004, **98**, 903.
7. F. Neese, *WIREs Comput. Molec. Sci.*, 2012, **2**, 73.
8. (a) A. D. Becke, *J. Chem. Phys.*, 1988, **84**, 4524. (b) J. P. Perdew, *Phys. Rev. B,* 1986, **33**, 8822.
9. D. A. Pantazis, X.-Y. Chen, C. R. Landis and F. Neese, *J. Chem. Theory Comput.*, 2008, **4**, 908.
10. (a) E. van Lenthe, J. G. Snijders and E. J. Baerends, *J. Chem. Phys.*, 1996, **105**, 6505. (b) E. van Lenthe, A. van der Avoird and P. E. S. Wormer, *J. Chem. Phys.*, 1998, **108**, 4783. (c) J. H. van Lenthe, S. Faas and J. G. Snijders, *Chem. Phys. Lett.*, 2000, **328**, 107.
11. C. J. van Wüllen, *J. Chem. Phys.*, 1998, **109**, 392.
12. A. Klamt and G. Schüüramann, *J. Chem. Soc., Perkin Trans. 2*, 1993, 799.
13. (a) P. Pulay, *Chem. Phys. Lett.*, 1980, **73**, 393. (b) P. Pulay, *J. Comput. Chem.*, 1982, **3**, 556.
14. (a) J. P. Perdew, K. Burke and M. Ernzerhof, *Phys. Rev. Lett.*, 1996, **77**, 3865. (b) C. Adamo and V. Barone, *J. Chem. Phys.*, 1999, **110**, 6158.
15. (a) F. Neese, F. Wennmohs, A. Hansen and U. Becker, *Chem. Phys.*, 2009, **356**, 98. (b) R. Izsák and F. Neese, *J. Chem. Phys.*, 2011, **135**, 144105.
16. (a) F. Neese, *J. Chem. Phys.*, 2001, **115**, 11080. (b) F. Neese, *J. Chem. Phys.*, 2003, **118**, 3939.
17. *Molekel*, Advanced Interactive 3D-Graphics for Molecular Sciences, Swiss National Supercomputing Center. https://ugovaretto.github.io/molekel/
18. S. R. Hartmann and E. L. Hahn, *Phys. Rev.*, 1962, **128**, 2042.
